# Effects of Enhanced External Counterpulsation With Different Sequential Levels on Lower Extremity Hemodynamics

**DOI:** 10.3389/fcvm.2021.795697

**Published:** 2021-12-24

**Authors:** Yahui Zhang, Yujia Zhang, Yinfen Wang, Xiuli Xu, Jing Jin, Xiaodong Zhang, Wei Zhang, Wenbin Wei, Chubin Zhong, Guifu Wu

**Affiliations:** ^1^Department of Cardiology, The Eighth Affiliated Hospital of Sun Yat-sen University, Shenzhen, China; ^2^NHC Key Laboratory of Assisted Circulation, Sun Yat-sen University, Guangzhou, China; ^3^Guangdong Innovative Engineering and Technology Research Center for Assisted Circulation, Shenzhen, China; ^4^Department of Physical Education, Nanjing University of Finance and Economics, Nanjing, China; ^5^College of Computer, Jilin Normal University, Siping, China; ^6^Department of Cardiac Ultrasound, The Eighth Affiliated Hospital, Sun Yat-sen University, Shenzhen, China

**Keywords:** enhanced external counterpulsation, lower extremity arteries, blood flow, hemodynamic responses, sequential level

## Abstract

**Objective:** This study aimed to investigate acute hemodynamics of lower extremities during enhanced external counterpulsation with a three-level sequence at the hips, thighs, and calves (EECP-3), two-level sequence at the hips and thighs (EECP-2), and single leg three-level sequence (EECP-1).

**Methods:** Twenty healthy volunteers were recruited in this study to receive a 45-min EECP intervention. Blood flow spectrums in the anterior tibial artery, posterior tibial artery, and dorsalis pedis artery were imaged by Color Doppler ultrasound. Mean flow rate (FR), area, pulsatility index (PI), peak systolic velocity (PSV), end-diastolic velocity (EDV), mean flow velocity (MV), and systolic maximum acceleration (CCAs) were sequentially measured and calculated at baseline during EECP-3, EECP-1, and EECP-2.

**Results:** During EECP-3, PI, PSV, and MV in the anterior tibial artery were significantly higher, while EDV was markedly lower during EECP-1, EECP-2, and baseline (all *P* < 0.05). Additionally, ACCs were significantly elevated during EECP-3 compared with baseline. Moreover, FR in the anterior tibial artery was significantly increased during EECP-3 compared with baseline (*P* = 0.048). During EECP-2, PI and MV in the dorsalis pedis artery were significantly higher and lower than those at baseline, (both *P* < 0.05). In addition, FR was markedly reduced during EECP-2 compared with baseline (*P* = 0.028). During EECP-1, the area was significantly lower, while EDV was markedly higher in the posterior tibial artery than during EECP-1, EECP-2, and baseline (all *P* < 0.05). Meanwhile, FR of the posterior tibial artery was significantly reduced compared with baseline (*P* = 0.014).

**Conclusion:** Enhanced external counterpulsation with three-level sequence (EECP-3), EECP-2, and EECP-1 induced different hemodynamic responses in the anterior tibial artery, dorsalis pedis artery, and posterior tibial artery, respectively. EECP-3 acutely improved the blood flow, blood flow velocity, and ACCs of the anterior tibial artery. In addition, EECP-1 and EECP-2 significantly increased the blood flow velocity and peripheral resistance of the inferior knee artery, whereas they markedly reduced blood flow in the posterior tibial artery.

## Introduction

Enhanced external counterpulsation (EECP) is a noninvasive treatment for patients with cardiovascular disease (1–3). Studies have also reported that EECP not only alleviates symptoms of angina and reduces myocardial ischemia (4–6) but that it is also beneficial to peripheral vascular function (7–9). Braith et al. found that EECP significantly increases peripheral artery flow-mediated dilation (FMD) and promotes endothelial-derived vasoactive agents (9). Nichols et al. demonstrated that EECP reduces arterial stiffness and improves wave reflection characteristics (7). Zhang et al. also found that EECP decreases resistance index in the common carotid artery (10). Gurovich et al. demonstrated that EECP-induced blood flow patterns improve endothelial function in peripheral muscular conduit arteries (11). Avery et al. also found that EECP significantly elevates peak limb blood flow and improves endothelium-dependent vasodilation in calf resistance arteries (12).

However, there were some controversial studies. Werner et al. found that EECP significantly reduces flow volumes of the posterior tibial artery (13). Dockery *et al*. demonstrated that EECP cannot reduce arterial stiffness (14). It showed that EECP is unlikely to be influenced by the alterations in mechanical properties of the arterial wall (14). Martin *et al*. reported that EECP does not significantly improve resistance arterial function in the calf (15). Whether EECP may provide vascular medicine to the peripheral arterial tree needed to be verified (12). In addition, Hashemi *et al*. found that endothelial function is not significantly improved after EECP intervention (16). Meanwhile, we found that peripheral hemodynamic responses induced by EECP highlighted personalized plans in patients with different cardiovascular risk factors (10). Furthermore, our study demonstrated that EECP creates different responses of vascular and blood flow characteristics in the carotid and peripheral arteries. More importantly, beneficial effects on the inner diameter, blood flow velocity, resistance index, and blood flow after 45-min-EECP were shown only in patients with coronary artery disease (17).

Based on the above-mentioned issues, Buschmann et al. proposed an improved treatment for patients with peripheral vascular disease (PAD), individual shear rate therapy (ISRT), like EECP-2, which includes two cuffs wrapped around the hip and thighs for ensuring adequate calf perfusion compared to EECP-3 with the three-cuff system (18–20). It is evaluated with real-time Doppler-derived variables of calf perfusion during counterpulsation (18). Studies have reported that ISRT improves endothelial function and increases lower limb walking distance (19). A study showed that ISRT can improve the degree of peripheral arteriosclerosis, increase exercise capacity, and reduce arterial blood pressure (21). However, a study reported that ISRT cannot reduce ankle brachial index and pulse wave velocity (22).

Currently, only few effective treatments for the improvement of lower limb hemodynamics have been proposed. Additionally, their clinical evidence is insufficient, and a related hemodynamic mechanism is not clear (13, 23, 24). Furthermore, to our knowledge, there is no effective treatment for lower limb stenosis in different parts (e.g., anterior tibial artery, posterior tibial artery, or dorsalis pedis artery). However, the pathogenesis of patients with PAD is too complex to investigate detailed hemodynamic changes (e.g., unilateral, bilateral, and multi-vascular). Therefore, based on the aforementioned EECP technology platform, we changed the sequential level, and set the monitoring scheme of EECP with a three-level sequence (EECP-3), the two-level sequence at the hips and thighs (EECP-2), and single leg three-level sequence (EECP-1). The ultrasonic blood flow spectrum of inferior knee arteries (anterior tibial artery, posterior tibial artery, and dorsalis pedis artery) was analyzed during EECP-3, EECP-2, and EECP-1. On the one hand, this study can clarify the acute responses of these treatment schemes on lower extremity hemodynamics. On the other hand, it may provide a theoretical basis of exercise physiology for lower extremity arteriosclerosis, that is, personalized treatment schemes for patients with a different lower limb arterial stenosis.

## Materials and Methods

### Subjects

Twenty young men (*n* = 20), ranging from 24 to 30 years old, were enrolled from the Health Examination Center of the Eighth Affiliated Hospital of Sun Yat-sen University (SYSU). All the participants were healthy and had no cardiovascular disease or related risk factors. Exclusion criteria consisted of exercising three times per week or more, known cardiovascular diseases, and contraindications of EECP and medication. Before the experiment, informed consent forms were signed by all the healthy participants. It was approved by the local medical ethics committee of the Eighth Affiliated Hospital of SYSU (2021-020-02).

### Experiment Protocol

**Figure 1:**
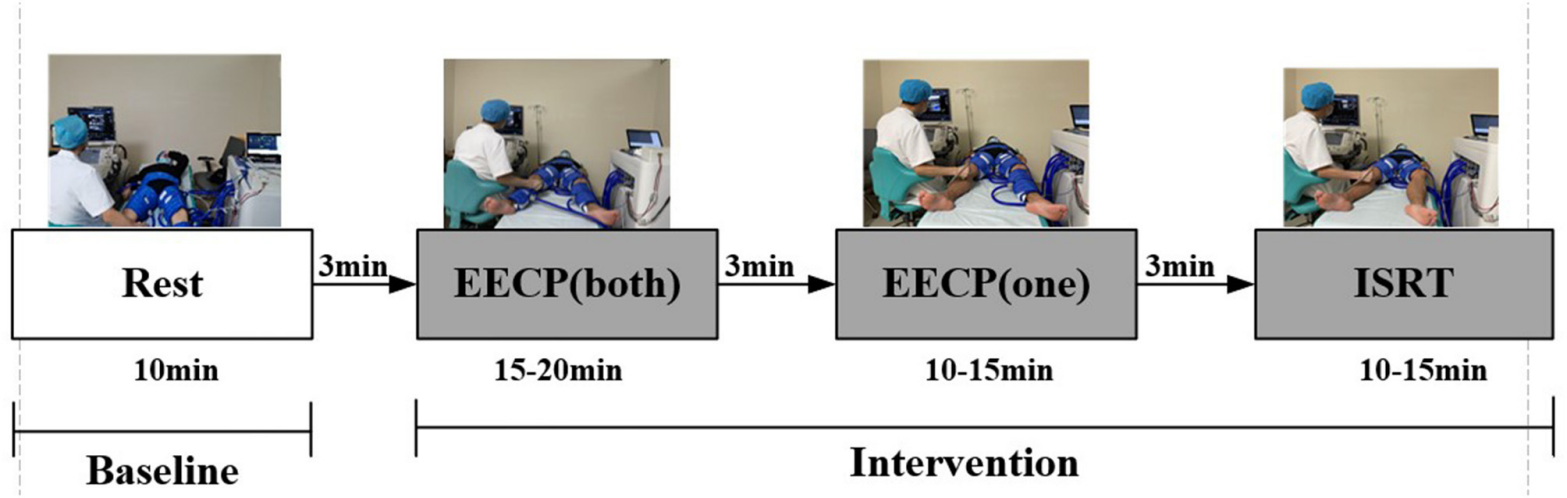
Flowchart of the whole experimental scheme.

Hemodynamic data on lower extremities were collected by echo investigation at 5 p.m. every day. Before the experiment, all the healthy participants were required to not eat any food or drink alcohol or caffeine, and avoid EECP or exercise for at least 24 h prior to measurements. The flowchart of the whole experimental scheme is illustrated in Figure 1. The baseline measurements were performed for each group in the supine position after 10 min of relaxation. Both blood pressure and heart rate were measured at baseline.

All the subjects first received EECP-3 intervention with the PSK P-ECP/TM Oxygen Saturation Monitoring EECP Instrument (Made in Chongqing, China). These healthy participants lay supine on the treatment bed with their legs and buttocks wrapped in cuffs, which were sequentially inflated from the lower thigh to the upper thigh and buttocks at the beginning of the diastolic phase, followed by quick, simultaneous deflation of all the cuffs just prior to the onset of systole. Second, all the healthy participants were conducted EECP-1 treatment. They lay supine on the treatment bed with one leg and buttocks wrapped in cuffs. Finally, EECP-2 was performed with no cuffs in the calves. The ultrasonic blood flow spectrum of inferior knee arteries (anterior tibial artery, posterior tibial artery, and dorsalis pedis artery) was monitored in each stage. A color Doppler ultrasound device (Toshiba Aplio 500 TUS-A500; Toshiba, Japan) was used to measure the hemodynamic information at baseline, during EECP- 3, EECP-2, and EECP-1.

### Parameter Calculation

Parameters, namely, PSV, end-diastolic velocity (EDV), mean flow velocity (MV), pulsatility index (PI), area, flow rate (FR), and systolic maximum acceleration (CCAs) were continuously recorded for 5 s, and then were calculated for mean value.

The mean PI of all the arteries was calculated as:


(1) $$\begin{array}{r} PI=\frac{PSV-EDV}{TAMAX} \end{array}$$

where $TAMAX=\frac{VTI}{T}$

Flow rate (FR) was calculated from vessel diameter, cardiac period, and velocity-time integral as:


(2) $$\begin{array}{r} FR=\frac{S\times VTI}{T} \end{array}$$

where VTI is the averaged velocity-time integral, and T is the averaged cardiac cycle time. S is a vascular area.

### Statistical Analysis

All the variables were the mean value of the area under the envelope curve in a cardiac cycle. Results are shown as means ± SD. Normal distribution for all the lower limb hemodynamic variables was evaluated by the Kolmogorov-Smirnov test (at least one test *P* > 0.05). Basic characteristics were determined by descriptive analysis. A repeated ANOVA comparing the parameters of inferior knee arteries (anterior tibial artery, posterior tibial artery, and dorsalis pedis artery) was performed at baseline, during EECP-3, EECP-2, and EECP-1. Additionally, a one-way ANOVA comparing hemodynamic variables among the three arteries was performed. Fisher's least significant difference test was conducted as a *post-hoc* analysis. All the statistical tests were conducted with SPSS version 20.0 (IBM SPSS Statistics, United States), and *p* < 0.05 was taken as a measure of statistical significance.

## Results

The baseline information, including age, gender, height, weight, risk factors (smoking, drinking, family history and sleep disorders, and exercise habits) were shown as below at resting conditions before EECP intervention (Table 1).

**Table 1:** Basic characteristics of the participants.

**Characteristics**	**Subjects (*n* = 20)**
Age, year	25.51 ± 2.28
Height, cm	173.20 ± 4.74
Weight, kg	66.83 ± 7.38
Smoking	0
Drinking	0
Exercise habits, *n* (%)	6 (30)
Sleep disorders, *n* (%)	5 (25)
Family history, *n* (%)	3 (15)
SBP (mmHg)	118.80 ± 7.92
DBP (mmHg)	72.35 ± 4.52
HR (bpm)	64.75 ± 8.93

**Figure 2:**
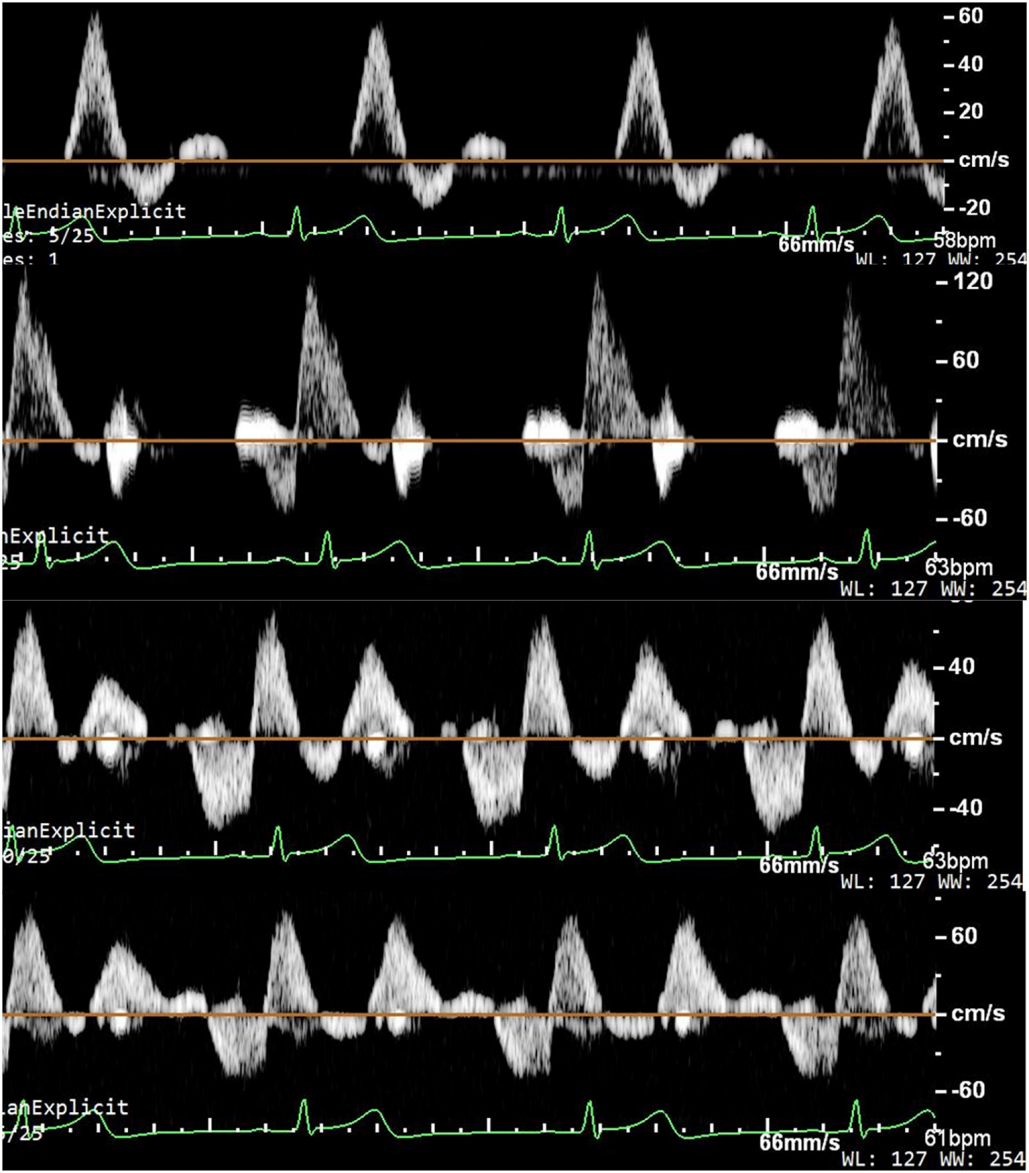
Ultrasound pictures and Doppler spectrum of the anterior tibial artery at baseline, during EECP-3, EECP-1, and EECP-2.

**Figure 3:**
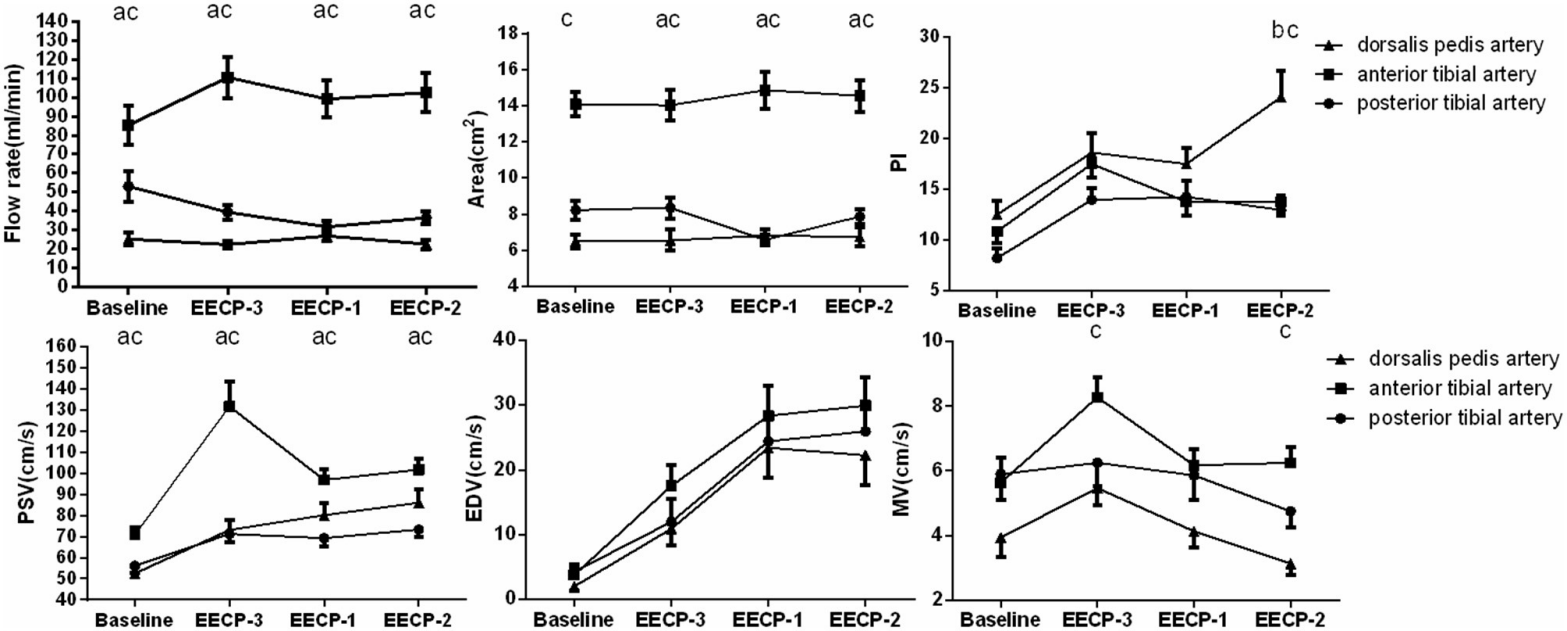
Significant changes in the anterior tibial artery, posterior tibial artery, and dorsalis pedis artery at baseline, during EECP-3, EECP-2, and EECP-1. a. significant differences between the anterior tibial artery and posterior tibial artery; b. significant difference between dorsalis pedis artery and posterior tibial artery; c. significant differences between the anterior tibial artery and dorsalis pedis artery.

Original ultrasonic pictures and the Doppler spectrum of the anterior tibial artery at baseline, during EECP-3, EECP-2 and EECP-1, are illustrated in Figure 2. Different hemodynamic responses (e.g., blood flow, blood flow velocity, and PI) among the anterior tibial artery, posterior tibial artery, and dorsalis pedis artery are shown in Figure 3. FR, area, and PSV in the anterior tibial artery were significantly higher than in the other arteries (all *P* < 0.01). MV of the anterior tibial artery was also significantly higher than that of the other arteries during EECP-3 and EECP-2 (both *P* < 0.01). However, PI of the dorsalis pedis artery was significantly higher than that of the other arteries during EECP-2 (both *P* < 0.01).

**Figure 4:**
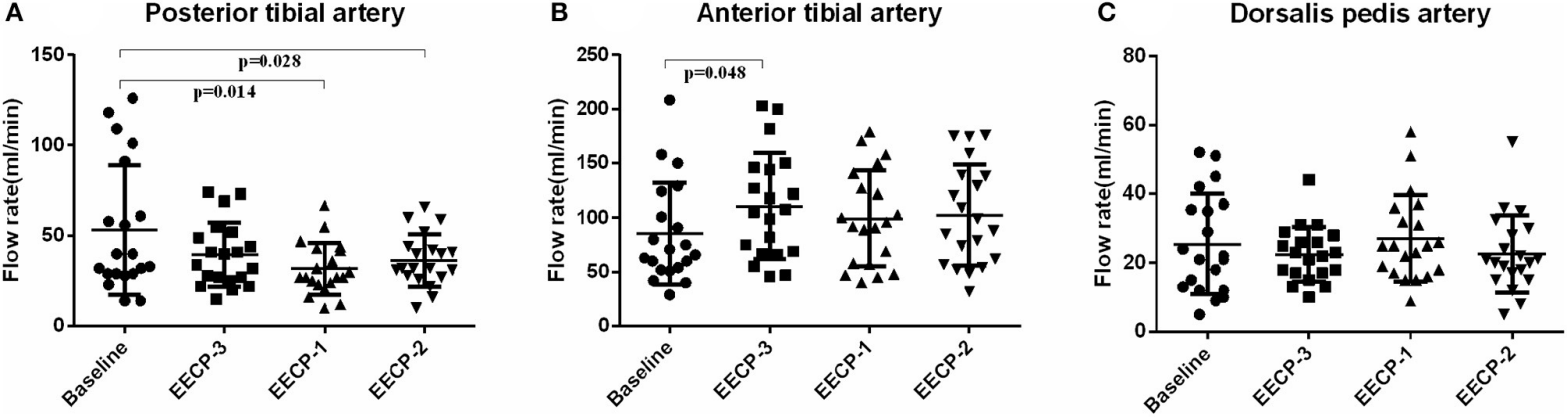
Effect of EECP-3, EECP-2 and EECP-1 on the flow rate (FR) of posterior tibial artery **(A)**, anterior tibial artery **(B)** and dorsalis pedis artery **(C)**.

**Figure 5:**
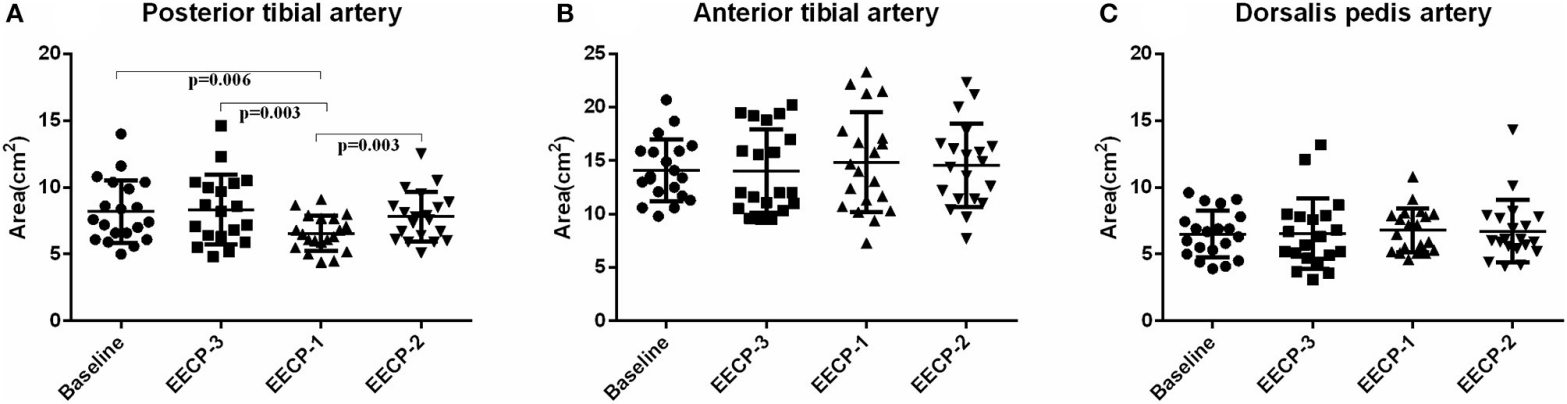
Effect of EECP-3, EECP-2 and EECP-1 on the area of posterior tibial artery **(A)**, anterior tibial artery **(B)** and dorsalis pedis artery **(C)**.

**Figure 6:**
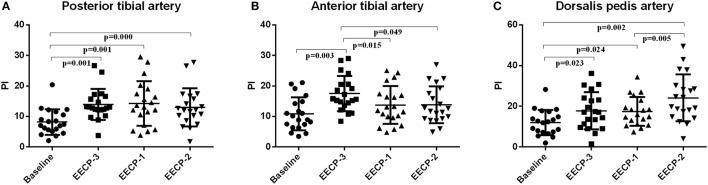
Effect of EECP-3, EECP-2 and EECP-1 on the pulsatility index (PI) of posterior tibial artery **(A)**, anterior tibial artery **(B)** and dorsalis pedis artery **(C)**.

**Figure 7:**
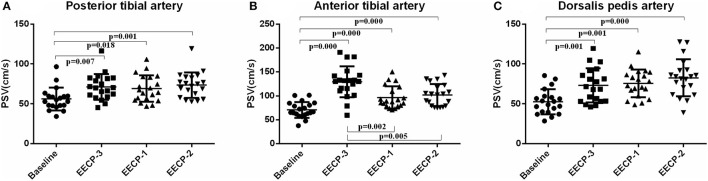
Effect of EECP-3, EECP-2 and EECP-1 on the peak systolic velocity (PSV) of posterior tibial artery **(A)**, anterior tibial artery **(B)** and dorsalis pedis artery **(C)**.

**Figure 8:**
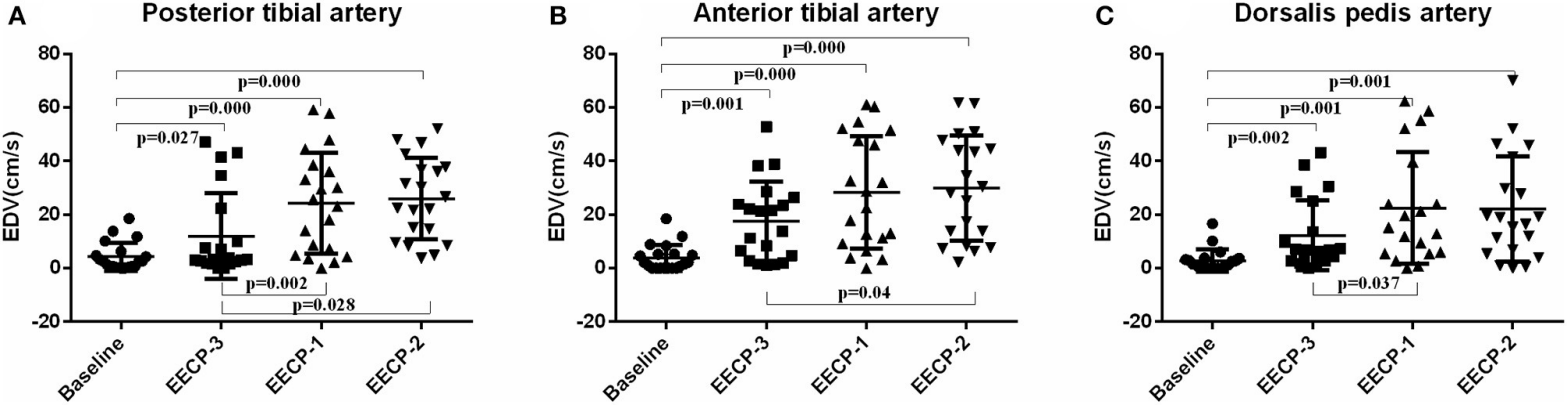
Effect of EECP-3, EECP-2 and EECP-1 on the end-diastolic velocity (EDV) of posterior tibial artery **(A)**, anterior tibial artery **(B)** and dorsalis pedis artery **(C)**.

**Figure 9:**
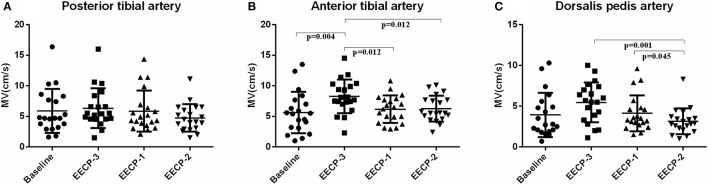
Effect of EECP-3, EECP-2 and EECP-1 on the mean flow velocity (MV) of posterior tibial artery **(A)**, anterior tibial artery **(B)** and dorsalis pedis artery **(C)**.

**Figure 10:**
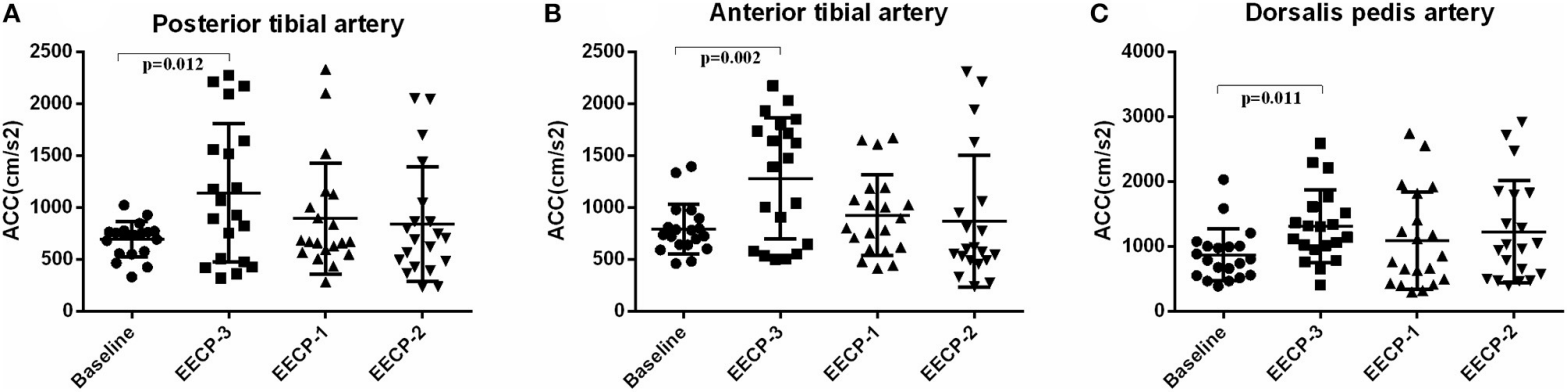
Effect of EECP-3, EECP-2 and EECP-1 on the systolic maximum acceleration (CCAs) of posterior tibial artery **(A)**, anterior tibial artery **(B)** and dorsalis pedis artery **(C)**.

Additionally, in order to clearly show hemodynamic response in each participant, the effects of EECP on the hemodynamic variables varied in each subject are illustrated in Figures 4–**10** and summarized in Supplementary Table 2.

### Blood Flow

Flow rate (FR) of the posterior tibial artery was significantly decreased during EECP-2 and EECP-1 compared with baseline (both *P* < 0.05, Figure 4A). However, FR of the anterior tibial artery was significantly increased during EECP-3 (*P* =0.048, Figure 4B). FR of the dorsalis pedis artery during EECP-2 was higher than that during EECP-3. In addition, the area, which is used to calculate FR in the posterior tibial artery during EECP-1, was significantly lower than that at baseline, during EECP-3 and EECP-2 (all *P* < 0.01, Figure 5A). However, there was no significant difference in the area of the anterior tibial artery and dorsalis pedis artery at baseline, during EECP-3, EECP-1, and EECP-2 (all *P* > 0.05, Figures 5B,C).

### Pulsatility Index

The pulsatility index (PI) of the posterior tibial artery during EECP-3, EECP-2, and EECP-1 was significantly higher than the baseline (all *P* < 0.01, Figure 6A). PI of the anterior tibial artery was also significantly higher during EECP-3 than during EECP-1 (*P* = 0.015) and EECP-2 (*P* = 0.049). Moreover, PI of the dorsalis pedis artery was markedly higher during EECP-2 than during EECP-1 (*P* = 0.003, Figure 6).

### Blood Flow Velocity

Peak systolic velocity (PSV) in the inferior knee artery was significantly increased during EECP-3, EECP-2, and EECP-1 compared with baseline (all *P* < 0.01, Figure 7). In addition, PSV of the anterior tibial artery was significantly higher during EECP-3 than during EECP-1 (*P* = 0.002) and EECP-2 (*P* = 0.005).

Compared with baseline, EDV of the inferior knee artery was significantly increased during EECP-3, EECP-2, and EECP-1 (all *P* < 0.01, Figure 8). EDV of the posterior tibial artery and dorsalis pedis artery during EECP-1 was higher than during EECP-3 (both *P* < 0.01, Figure 8A). Additionally, EDV of the anterior tibial artery and the posterior tibial artery was also significantly higher during EECP-2 than during EECP-3 in this study (both *P* < 0.01, Figures 8A,B).

Mean flow velocity (MV) of the anterior tibial artery during EECP-3 was markedly higher than the baseline (*P* = 0.004), during EECP-1 (*P* = 0.012) and EECP-2 [(*P* = 0.012), Figure 9], while MV of the dorsalis pedis artery was markedly lower during EECP-2 than during EECP-3 (*P* = 0.001) and EECP-1 (*P* = 0.045, Figure 9C). There was no significant change in MV of the posterior tibial artery in each stage (all *P* > 0.05, Figure 9).

### Systolic Maximum Acceleration (CCAs)

During EECP-3, CCAs in the anterior tibial artery, posterior tibial artery, and dorsalis pedis artery was significantly increased compared with the baseline (all *P* < 0.01, Figure 10). However, there was no significant difference in CCAs at baseline during EECP-1 and EECP-2 (all *P* > 0.05).

## Discussion

This study was designed to investigate lower extremity hemodynamics during EECP-3, EECP-2, and EECP-1, and determine acute hemodynamic effects on ultrasonic blood flow spectrum data. The major findings in this study are 2-fold: first, EECP-3 immediately improved the blood flow, blood flow velocity, and ACCs of the anterior tibial artery; second, EECP-1 and EECP-2 significantly increased the blood flow velocity and peripheral resistance of the inferior knee artery, whereas they markedly reduce the blood flow in the posterior tibial artery.

In this study, we found that EECP significantly increased the FR of the anterior tibial artery and that the FR of the anterior tibial artery was significantly higher than that of the other arteries. Few studies have investigated the acute effects of EECP on lower extremity hemodynamics. Studies have reported that inflation of the EECP-3 cuffs creates the high-pressure retrograde blood flow in the femoral arteries (11). Cai et al. (23) also found that EECP could improve blood circulation in the lower extremities by combining animal and human experiments. In addition, our previous study has shown that EECP increases the FR of the femoral artery (17). Meanwhile, Avery et al. found that EECP significantly increased FR and improved endothelium-dependent vasodilation in calf resistance arteries. EECP may provide a kind of “massage” on the peripheral function, starting with the elastic conduit arteries, muscular conduit arteries, and extending to the skeletal muscle resistance arteries (12). The diastolic inflation/systolic deflation sequence of EECP-3 results in diastolic augmentation/systolic unloading and leads to increased blood flow (8, 25).

However, our results are inconsistent, at least in part with the above findings. After changing the limb cuffs, FR in the posterior tibial artery was significantly decreased during both EECP-2 and EECP-1. Werner et al. found that the FR of the posterior tibial artery decreased to 69 ± 23% during EECP (13). Studies found that reduced peripheral flow during EECP may have similar physiologic effects like an exercise in patients with symptomatic PAD. More importantly, it is contributed by changes in MV and PI (13). PI of the posterior tibial and dorsalis pedis arteries markedly increased compared with baseline. In addition, MV of the anterior tibial artery during EECP-3 was markedly higher than the baseline (*P* =0.004), during EECP-1 (*P* =0.012) and EECP-2 (*P* = 0.012), while MV of the dorsalis pedis artery during EECP-2 was markedly lower than during EECP-3 (*P* = 0.001) and EECP-1 (*P* = 0.045). Werner et al. assessed the blood flow velocity of the posterior tibial artery during EECP (13). They found a significant increase in the MV and PI, showing a marked increase in retrograde blood flow. EECP creates a second diastolic pulse wave in all arterial vessels (26).

In this study, during EECP-2, PI and MV of the dorsalis pedis artery were significantly increased and decreased, respectively. In addition, PI of the dorsalis pedis artery was significantly higher than that of the other arteries during EECP-2. The findings of this study support a previous study, which show that the PI of the posterior tibial artery has a 4-fold increase (13). Studies have reported that PI is an important indicator of peripheral resistance (27–29), which can be associated with postural changes, physiologic fluctuations, and vascular disease (30–32). A second diastolic pulse wave in the lower extremity artery was created by EECP-2. Additionally, changes in peripheral flow patterns during EECP-2 were also characterized by elevated PI.

Studies have also reported that regulation of FR is associated with vascular diameters and blood flow velocity (33). In this study, blood flow velocity, like PSV and EDV, in the inferior knee artery significantly increased. Few studies investigated the PSV of the inferior knee artery during EECP. Zhang et al. also found that vascular diameter and PSV were significantly increased after EECP intervention. Besides that, the acceleration of systolic peak velocity also increased. A study showed that the ACC of systolic peak velocity in the lower limbs is an effective marker of peripheral artery disease (34, 35). Cai et al. observed a 1.2-fold increase in femoral artery retrograde blood flow velocity (23). Based on a porcine EECP model, Zhan*g et al*. demonstrated that blood flow velocity and wall shear stress of the peripheral artery increased by 1.3- and 2.1-fold, respectively, during EECP (8). Additionally, EECP increases flow pulsatility and shear stress (13). Gurovich et al. also found that EECP significantly increased retrograde shear stress and retrograde-turbulent FR in the femoral artery (11). The mechanism responsible for this phenomenon is increased endothelial shear stress, which leads to vascular anti-inflammatory changes in human umbilical vein endothelial cells (36).

Moreover, they showed that changes in shear rate, led by femoral artery vascular tone, elicited increased femoral baseline diameter after EECP intervention (11). Werner et al. reported that the diameter of the posterior tibial artery was significantly decreased after EECP intervention (13). Sonka et al. demonstrated that femoral peak diameters were regarded as the single peak diameter investigated during the plateau phase after cuff deflation (37). Dopheide et al. found that femoral artery diameter and vascular shear stress were significantly increased after supervised exercise training (38). Nevertheless, in this study, the area of the posterior tibial artery was significantly reduced during EECP-1, while there was no significant difference in the area during EECP-3 and EECP-2. Studies have reported that both flow- and pressure-induced forces play an important role in vessel wall diameter (39–41). Decreased area induced by EECP-1 may be associated with different changes of pressure in both legs.

### Limitations

Some limitations of this study should be emphasized. First, in order to explore physiological changes in lower limb hemodynamics, the participants in this study are healthy, young individuals, whereas EECP is normally prescribed for patients with cardiovascular disease. Second, we just investigate the acute effects of EECP on the lower extremity vascular parameters due to obtaining each immediate response of hemodynamics. Finally, we did not measure lower limb FMD, so we were not able to assess endothelial function after EECP in these arteries.

### Future Direction

Further studies that will investigate EECP with different sequence level-induced lower limb hemodynamics in patients with PAD are appropriate. In addition, the long-term effects of EECP with different sequence levels on lower limb vascular function in patients with PAD will be explored in the future.

## Conclusion

This study demonstrated that sensitive parameters in the anterior tibial artery, dorsalis pedis artery, and posterior tibial artery are highlighted during EECP-3, EECP-2, and EECP-1, respectively. EECP-3 immediately improved the blood flow, blood flow velocity, and ACCs of the anterior tibial artery. EECP-1 mainly regulated the hemodynamic indexes of the posterior tibial artery, such as FR and area. In contrast, EECP-2 significantly regulated the PI and MV of the dorsalis pedis artery. This study will be beneficial to realize the personalized and precise treatment of PAD with external counterpulsation. EECP-3 may be recommended for patients with anterior tibial artery stenosis. EECP-2 may be recommended to improve lower artery hemodynamics of patients with dorsal foot artery stenosis. On the contrary, EECP-2 and EECP-1 may be not suitable for the treatment of the posterior tibial artery.

## Data Availability Statement

The original contributions presented in the study are included in the article/Supplementary Material, further inquiries can be directed to the corresponding authors.

## Ethics Statement

The studies involving human participants were reviewed and approved by the Local Medical Ethics Committee of The Eighth Affiliated Hospital of SYSU (2021-020-02). The patients/participants provided their written informed consent to participate in this study.

## Author Contributions

YuZ, CZ, and GW proposed the scientific problems. YuZ and CZ designed the experiments. YuZ, YaZ, WW, YW, XX, and JJ collected the experimental data. YuZ, WZ, and XZ processed and calculated the data. YuZ conducted the statistical analysis and wrote the draft manuscript. CZ and GW contributed to the revision and final version of the manuscript. All authors contributed to the article and approved the submitted version.

## Funding

This study was, in part, supported by the National Key Research and Development Program of China (No. 2020YFC2004400), and the National Natural Science Foundation of China [Grant Nos. 819770367 and 81670417]. Part of this research was supported by Shenzhen Key Clinical Discipline Funds (ZDXKJF-01002).

## Conflict of Interest

The authors declare that the research was conducted in the absence of any commercial or financial relationships that could be construed as a potential conflict of interest.

## Publisher's Note

All claims expressed in this article are solely those of the authors and do not necessarily represent those of their affiliated organizations, or those of the publisher, the editors and the reviewers. Any product that may be evaluated in this article, or claim that may be made by its manufacturer, is not guaranteed or endorsed by the publisher.
