# Supplementary material for: Effects of Enhanced External Counterpulsation With Different Sequential Levels on Lower Extremity Hemodynamics
Source: Front Cardiovasc Med. 2021 Dec 24;8:795697. doi: 10.3389/fcvm.2021.795697 (PMC8739776; doi:10.3389/fcvm.2021.795697)
Supplement: Supplementary file 2 [file Table_2.DOCX]

**Table 2 Effect of EECP-3, EECP-1 and EECP-2 on the lower extremity hemodynamics of anterior tibial artery, posterior tibial artery and dorsalis pedis artery.**

| **Variables** | **Posterior tibial artery** | **Condition** | **P**  **value** | **Anterior tibial artery** | **Condition** | **P value** | **Dorsalis pedis artery** | **Condition** | **P value** |
| --- | --- | --- | --- | --- | --- | --- | --- | --- | --- |
| **FR** | 53.15±35.87 |  |  | 85.23±46.56 | 1-2 | 0.048 | 25.43±14.54 |  |  |
|  | 39.50±17.81 |  |  | 110.55±48.77 |  |  | 22.50±7.97 |  |  |
|  | 31.75±14.36 | 1-3 | 0.014 | 99.15±44.15 |  |  | 27.05±12.54 |  |  |
|  | 36.55±14.55 | 1-4 | 0.028 | 102.53±46.46 |  |  | 22.68±11.24 |  |  |
|  |  |  |  |  |  |  |  |  |  |
| **S** | 8.22±2.41 | 1-3 | 0.006 | 14.09±2.88 |  |  | 6.50±1.74 |  |  |
|  | 8.34±2.60 | 2-3 | 0.003 | 14.03±3.89 |  |  | 6.54±2.64 |  |  |
|  | 6.57±1.36 | 3-4 | 0.003 | 14.87±4.65 |  |  | 6.80±1.65 |  |  |
|  | 7.86±1.88 |  |  | 14.55±3.89 |  |  | 6.73±2.32 |  |  |
|  |  |  |  |  |  |  |  |  |  |
| **PI** | 8.19±4.21 | 1-2 | 0.001 | 10.84±5.37 | 1-2 | 0.003 | 12.48±5.92 | 1-2 | 0.023 |
|  | 13.95±5.06 | 1-3 | 0.001 | 17.48±5.73 | 2-3 | 0.015 | 18.61±8.49 | 1-3 | 0.024 |
|  | 14.23±7.33 | 1-4 | 0 | 13.75±6.22 | 2-4 | 0.049 | 17.48±7.13 | 1-4 | 0.002 |
|  | 12.94±6.27 |  |  | 13.78±6.04 |  |  | 24.07±11.83 | 3-4 | 0.005 |
|  |  |  |  |  |  |  |  |  |  |
| **PSV** | 56.00±14.16 | 1-2 | 0.007 | 70.79±16.45 | 1-2 | 0 | 52.46±15.68 | 1-4 | 0 |
|  | 71.21±16.22 | 1-3 | 0.018 | 131.76±52.18 | 1-3 | 0 | 73.19±21.50 | 1-2 | 0.001 |
|  | 69.22±16.56 | 1-4 | 0.001 | 96.80±23.17 | 1-4 | 0 | 80.20±26.00 | 1-3 | 0.001 |
|  | 73.39±16.02 |  |  | 101.74±22.69 | 2-3 | 0.002 | 86.07±28.23 |  |  |
|  |  |  |  |  | 2-4 | 0.005 |  |  |  |
| **EDV** | 4.27±5.26 | 1-3 | 0 | 3.77±4.93 | 1-2 | 0.001 | 2.01±2.67 | 1-2 | 0.002 |
|  | 11.99±16.05 | 1-4 | 0 | 17.52±14.73 | 1-3 | 0 | 10.92±11.56 | 1-3 | 0.001 |
|  | 24.41±18.84 | 2-4 | 0.002 | 28.36±21.03 | 1-4 | 0 | 23.41±21.02 | 1-4 | 0.001 |
|  | 25.96±15.23 | 1-2 | 0.027 | 29.93±19.58 | 2-4 | 0.04 | 22.26±20.73 | 2-3 | 0.037 |
|  |  | 2-3 | 0.028 |  |  |  |  |  |  |
| **MDV** | 5.90±3.60 |  |  | 5.64±3.40 | 1-2 | 0.006 | 3.94±2.71 | 2-4 | 0.001 |
|  | 6.25±3.25 |  |  | 8.28±2.75 | 2-3 | 0.012 | 5.47±2.45 | 3-4 | 0.045 |
|  | 5.86±3.35 |  |  | 6.18±2.24 | 2-4 | 0.012 | 4.13±2.19 |  |  |
|  | 4.75±2.24 |  |  | 6.25±2.11 |  |  | 3.13±1.59 |  |  |
|  |  |  |  |  |  |  |  |  |  |
| **ACC** | 695.38±167.68 | 1-2 | 0.012 | 843.81±405.54 | 1-2 | 0.018 | 867.55±402.29 | 1-2 | 0.011 |
|  | 1141.13±669.13 |  |  | 1483.32±970.49 |  |  | 1310.11±563.36 |  |  |
|  | 896.05±534.65 |  |  | 1327.46±1634.92 |  |  | 1090.68±749.97 |  |  |
|  | 838.72±552.79 |  |  | 1249.99±1403.31 |  |  | 1222.40±788.87 |  |  |

**Note: FR: Mean flow rate; PI: pulsatility index, PSV: Peak systolic velocity, EDV: end-diastolic velocity, MV: mean flow velocity, and CCAs: systolic maximum acceleration. Condition 1, baseline; Condition 2, EECP-3; Condition 3, EECP-1; Condition 4, EECP-2.**
