# Supplementary material for: Effects of Enhanced External Counterpulsation With Different Sequential Levels on Lower Extremity Hemodynamics
Source: Front Cardiovasc Med. 2021 Dec 24;8:795697. doi: 10.3389/fcvm.2021.795697 (PMC8739776; doi:10.3389/fcvm.2021.795697)
Supplement: Supplementary file 3 [file Table_3.DOCX]

**Effects of Enhanced External Counterpulsation with Different Sequential Levels on Lower Extremity Hemodynamics**

**Yahui Zhang^1,2,3^, Yujia Zhang^1,2,3^, Yinfen Wang^1^, Xiuli Xu^1,2,3^, Jing Jin^1^, Xiaodong Zhang^4^, Wei Zhang^5^, Wenbin Wei^1^, Chubin Zhong^6*^, Guifu Wu^1,2,3*^**

^1^Department of Cardiology, the Eighth Affiliated Hospital of Sun Yat-sen University, Shenzhen 518033, China.

^2^NHC Key Laboratory of Assisted Circulation (Sun Yat-sen University), Guangzhou 510080, China

^3^Guangdong Innovative Engineering and Technology Research Center for Assisted Circulation, Shenzhen 518033, China.

^4^Department of Physical Education, Nanjing University of Finance and Economics, Nanjing, Jiangsu, 210023 China.

^5^College of Computer, Jilin Normal University, Siping, 136000, P. R. China.

^6^Department of Cardiac Ultrasound, The Eighth Affiliated Hospital, Sun Yat-sen University, Shenzhen, Guangdong, 518033 China.

***Correspondence:**Corresponding Author1: Guifu Wu

email: [wuguifu@mail.sysu.edu.cn](mailto:wuguifu@mail.sysu.edu.cn)

Corresponding Author2: Chubin Zhong

email: [838795654@qq.com](mailto:838795654@qq.com)

Keywords: enhanced external counterpulsation; lower extremity arteries; blood flow; hemodynamic responses; sequential level.

## Abstract

**Objective:** This study aimed to investigate acute hemodynamics of lower extremities during the Enhanced External Counterpulsation with three-level sequence at hips, thighs and calves (EECP-3), two-level sequence at hips and thighs (EECP-2) and single leg three-level sequence (EECP-1).

**Methods:** Twenty healthy volunteers were recruited into this study to receive 45min-

EECP intervention. Blood flow spectrums in the anterior tibial artery, posterior tibial artery and dorsalis pedis artery were imaged by Color Doppler ultrasound. Mean flow rate (FR), Area, pulsatility index (PI), Peak systolic velocity (PSV), end-diastolic velocity (EDV), mean flow velocity (MV) and systolic maximum acceleration (CCAs)

were sequentially measured and calculated at baseline, during EECP-3, EECP-1 and EECP-2.

**Results:** During EECP-3, PI, PSV and MV in the anterior tibial artery were significantly higher, while EDV was markedly lower than those during EECP-1, EECP-2 and baseline (all P<0.05). Additionally, ACCs was significantly elevated during EECP-3 compared with baseline. Moreover, FR in the anterior tibial artery was significantly increased during EECP-3 compared with baseline (P=0.048). During EECP-2, PI and MV in the dorsalis pedis artery was significantly higher and lower than those at baseline, respectively (both P<0.05). In addition, FR was markedly reduced during EECP-2 compared with baseline (P=0.028). During EECP-1, Area was significantly lower, while EDV was markedly higher in the posterior tibial artery than those during EECP-1, EECP-2 and baseline (all P<0.05). Meanwhile, FR of posterior tibial artery was significantly reduced compared with baseline (P=0.014).

**Conclusion:** EECP-3, EECP-2 and EECP-1 induced different hemodynamic responses in the anterior tibial artery, dorsalis pedis artery and posterior tibial artery, respectively.

EECP-3 creates acute improvement in the blood flow, blood flow velocity and ACCs of anterior tibial artery. In addition, EECP-1 and EECP-2 significantly increase blood flow velocity and peripheral resistance of inferior knee artery, whereas they markedly reduce blood flow in the posterior tibial artery.

## Introduction

Enhanced external counterpulsation (EECP) is a noninvasive treatment for patients with cardiovascular disease (Masuda et al., 2001; Fihn et al., 2012; Montalescot et al., 2013). Studies have also reported that EECP not only alleviates symptoms of angina and reduces myocardial ischemia (Wu et al., 2020; Caceres et al., 2021; Wu et al., 2021), but also is beneficial to peripheral vascular function (Nichols et al., 2006; Zhang et al., 2007; Braith et al., 2010a). Braith *et al.* found that EECP significantly increases peripheral artery flow-mediated dilation (FMD) and promotes endothelial-derived vasoactive agents (Braith et al., 2010b). Nichols *et al.* demonstrated that EECP reduces arterial stiffness and improves wave reflection characteristics (Nichols et al., 2006), Zhang *et al.* also found that EECP reduces resistance index of peripheral vascular (Zhang et al., 2021b). Gurovich *et al.* demonstrated that EECP-induced blood flow patterns improve endothelial function in peripheral muscular conduit arteries (Gurovich and Braith, 2013). Avery *et al.* also found that EECP significantly elevated peak limb blood flow and improves endothelium-dependent vasodilation in calf resistance arteries (Avery et al., 2014).

However, there were some controversial studies. Werner *et al.* found that EECP significantly reduces flow volumes of posterior tibial artery (Werner et al., 2007). Dockery *et al.* demonstrated that EECP cannot reduce arterial stiffness (Dockery et al., 2004). It showed that EECP is unlikely to be influenced on the alterations in mechanical properties of the arterial wall (Dockery et al., 2004). Martin *et al.* reported that EECP does not significantly improve resistance arterial function in the calf (Martin et al., 2014). Whether EECP may provide vascular medicine to the peripheral arterial tree needed to be verified (Avery et al., 2014). In addition, Hashemi *et al.* found endothelial function is not significantly improved after EECP intervention (Hashemi et al., 2008). Meanwhile, we found that peripheral hemodynamic responses induced by EECP highlighted personalized plans in patients with different cardiovascular risk factors (Zhang et al., 2021b). Furthermore, our study also demonstrated that EECP creates different responses of vascular and blood flow characteristics in carotid and peripheral arteries. More importantly, beneficial effects in inner diameter, blood flow velocity, resistance index, and blood flow after 45 min-EECP were shown only in the patients with coronary artery disease (Zhang et al., 2021a).

Based on above-mentioned issues, Buschmann *et al.* proposed an improved treatment for patients with peripheral vascular disease (PAD), Individual shear rate therapy (ISRT), like EECP-2, which includes two cuffs wrapped around the hip and thighs for ensuring adequate calf perfusion compared to EECP-3 with three-cuff system (Buschmann et al., 2010; Buschmann et al., 2016; Zietzer et al., 2017). It is evaluated by via real-time doppler-derived variables of the calf perfusion during counterpulsation (Buschmann et al., 2010). Studies have reported that ISRT improves endothelial function and increases lower limb walking distance (Buschmann et al., 2016). A study showed that ISRT can improve the degree of peripheral arteriosclerosis, increase exercise capacity and reduce arterial blood pressure (Picard et al., 2018). However, a study reported that ISRT cannot reduce ankle brachial index and pulse wave velocity (Picard et al., 2020).

Currently, few effective treatments for the improvement of lower limb hemodynamics were proposed. Additionally, their clinical evidence is insufficient, and related hemodynamic mechanism is not clear (Cai et al., 2000; Werner et al., 2007; Thakkar et al., 2010). Furthermore, to our knowledge, there is no effective treatment for lower limb stenosis in different parts (e.g. anterior tibial artery, posterior tibial artery or dorsalis pedis artery). However, pathogenesis of PAD patients is too complex to investigate detailed hemodynamic changes (e.g. unilateral, bilateral and multi-vascular). Therefore, based on aforementioned EECP technology platform, we changed sequential level, and set monitoring scheme of EECP with three-level sequence (EECP-3), two-level sequence at hips and thighs (EECP-2) and single leg three-level sequence (EECP-1). Ultrasonic blood flow spectrum of inferior knee artery (anterior tibial artery, posterior tibial artery and dorsalis pedis artery) were analyzed during EECP-3, EECP-2 and EECP-1. On the one hand, this study can clarify the acute responses of these treatment schemes on lower extremity hemodynamics; on the other hand, it may provide a theoretical basis of exercise physiology for lower extremity arteriosclerosis, that is, personalized treatment schemes for patients with different lower limb arterial stenosis.

## Materials and Methods

## Subjects

Twenty young men (n=20), ranging from 24 to 30 years old were enrolled from Health Examination Center of the Eighth Affiliated Hospital of Sun Yat-sen University (SYSU). All participants were healthy without cardiovascular disease or related risk factors. Exclusion criteria consists of exercising 3 times per week or more, known cardiovascular diseases and contraindications of EECP and medication. Before experiment, informed consent forms were signed by all the healthy participants. It was approved by the local medical ethics committee of the Eighth Affiliated Hospital of SYSU (2021-020-02).

## Experiment Protocol

Hemodynamic data at lower extremities were collected by echo investigation at 5pm every day. Before experiment, all healthy participants were required not to eat any food or drink alcohol, caffeine and avoid to EECP or exercise for at least 24 h prior to the measurements. The flowchart of whole experimental scheme was illustrated in Figure 1. The baseline measurements were performed for each group in the supine position after 10min relaxation. Both blood pressure and heart rate were measured at baseline.

All subjects first received EECP-3 intervention with the PSK P-ECP/TM Oxygen Saturation Monitoring EECP Instrument (Made in Chongqing, China). These healthy participants lay supine on the treatment bed with their legs and buttocks wrapped in cuffs, which were sequentially inflated from the lower thigh to the upper thigh and buttocks at the beginning of diastolic phase, followed by a quick, simultaneous deflation of all cuffs just prior to the onset of systole. Secondly, all healthy participants were conducted EECP-1 treatment. They lay supine on the treatment bed with one leg and buttocks wrapped in cuffs. Finally, EECP-2 was performed with no cuffs in the calves. Ultrasonic blood flow spectrum of inferior knee artery (anterior tibial artery, posterior tibial artery and dorsalis pedis artery) were monitored in each stage. Color Doppler Ultrasound (TOSHIBA APLIO 500 TUS-A500) was used to measure the hemodynamic information at baseline, during EECP-3, EECP-2 and EECP-1.

## Parameter calculation

Parameters, PSV, end-diastolic velocity (EDV), mean flow velocity (MV), pulsatility index (PI), Aera, flow rate (FR), and systolic maximum acceleration (CCAs) were continuously recorded for 5s, and then were calculated for mean value.

The mean PI of all arteries were calculated as:

$PI=\frac{PSV-EDV}{TAMAX}$, (1)

where $TAMAX=\frac{VTI}{T}$.

Flow rate (FR) was calculated from vessel diameter, cardiac period and the velocity-time integral as:

$FR=(S\times VTI)/T$ , (2)

where VTI is the averaged velocity-time integral, and T is the averaged cardiac cycle time. S is vascular area.

## Statistical analysis

All the variables were the mean value of area under the envelope curve in a cardiac cycle. Results are showed as means±sd. Normal distribution for all the lower limb hemodynamic variables was evaluated by the Kolmogorov-Smirnov test (at least one test P>0.05). Basic characteristics were conducted by the Descriptive Analysis. The repeated ANOVA comparing parameters of inferior knee artery (anterior tibial artery, posterior tibial artery and dorsalis pedis artery) were performed at baseline, during EECP-3, EECP-2 and EECP-1. Additionally, one-way ANOVA comparing hemodynamic variables among three arteries. Fisher’s least significant difference was conducted as *post-hoc* analysis. All statistical tests were conducted by SPSS version 20.0 (IBM SPSS Statistics, USA), and p<0.05 was taken as a measure of statistical significance.

## Results

The baseline information, including age, gender, height, weight, risk factors (smoking, drinking, family history and sleep disorders, and exercise habits were shown as below at resting conditions before EECP intervention (Table 1).

Original ultrasonic pictures and Doppler spectrum of anterior tibial artery at baseline, during EECP-3, EECP-2 and EECP-1 were illustrated in Figure 2. Different hemodynamic responses (e.g. blood flow, blood flow velocity and PI) among the anterior tibial artery, posterior tibial artery and dorsalis pedis artery were showed in Figure 3. FR, Area and PSV in the anterior tibial artery were significantly higher than in other arteries (all P<0.01). MV of anterior tibial artery was also significantly higher than that of other arteries during EECP-3 and EECP-2 (both P<0.01). However, PI of dorsalis pedis artery was significantly higher in dorsalis pedis artery than that of other arteries during EECP-2 (both P<0.01).

Additionally, in order to clearly show hemodynamic response in each participant, effects of EECP on the hemodynamic variables varied in each subject were illustrated in Figures 4 to 10 and summarized in S1 Table 2.

**Blood flow**

FR of posterior tibial artery was significantly decreased during EECP-2 and EECP-1 compared with baseline (both P<0.05, Figure 4(a)). However, FR of anterior tibial artery was significantly increased during EECP-3 (P=0.048, Figure 4(b)). FR of dorsalis pedis artery during EECP-2 was higher than that during EECP-3. In addition, Area which is used to calculate FR in the posterior tibial artery during EECP-1 was significantly lower than that at baseline, during EECP-3 and EECP-2 (all P<0.01, Figure 5(a)). However, there was no significant difference in Area of anterior tibial artery and dorsalis pedis artery at baseline, during EECP-3, EECP-1 and EECP-2 (all P>0.05, Figure 5(b and c)).

**Pulsatility index**

PI of posterior tibial artery during EECP-3, EECP-2 and EECP-1 was significantly higher compared with baseline (all P<0.01, Figure 6(a)). PI of anterior tibial artery was also significantly higher during EECP-3 than that during EECP-1 (P=0.015) and EECP-2 (P=0.049). Moreover, PI of dorsalis pedis artery was markedly higher during EECP-2 compared with EECP-1 (P=0.003, Figure 6(c)).

**Blood flow velocity**

PSV in the inferior knee artery was significantly increased during EECP-3, EECP-2 and EECP-1 compared with baseline (all P<0.01, Figure 7). In addition, PSV of anterior tibial artery was significantly higher during EECP-3 than that during EECP-1 (P=0.002) and EECP-2 (P=0.005).

Compared with baseline, EDV of inferior knee artery was significantly increased during EECP-3, EECP-2 and EECP-1 (all P<0.01, Figure 8). EDV of posterior tibial artery and dorsalis pedis artery during EECP-1 was higher than that during EECP-3 (both P<0.01, Figure 8(a)). Additionally, EDV of anterior tibial artery and posterior tibial artery was also significantly higher during EECP-2 than that during EECP-3 in this study (both P<0.01, Figure 8(a and b)).

MV of anterior tibial artery during EECP-3 was markedly higher than that at baseline (P=0.004), during EECP-1 (P=0.012) and EECP-2 ((P=0.012), Figure 9), while MV of dorsalis pedis artery was markedly lower during EECP-2 than that during EECP-3 (P=0.001) and EECP-1 (P=0.045, Figure 9(c)). There was no significantly change of MV in the posterior tibial artery at each stage (all P>0.05, Figure 9).

**Systolic maximum acceleration (CCAs)**

During EECP-3, CCAs in the anterior tibial artery, posterior tibial artery and dorsalis pedis artery was significantly increased compared with baseline (all P<0.01, Figure 10). However, there was no significant difference in CCAs at baseline, during EECP-1 and EECP-2 (all P>0.05).

## Discussion

The present study was designed to investigate lower extremity hemodynamics during EECP-3, EECP-2 and EECP-1, and determine the acute hemodynamic effects on ultrasonic blood flow spectrum data. The major findings in this study are twofold: first, EECP-3 creates immediate improvement in the blood flow, blood flow velocity and ACCs of anterior tibial artery; second, EECP-1 and EECP-2 significantly increase blood flow velocity and peripheral resistance of inferior knee artery, whereas they markedly reduce blood flow in the posterior tibial artery.

In the present study, we found that EECP significantly increased FR of anterior tibial artery, and FR in the anterior tibial artery was significantly higher than in other arteries. Few studies have investigated acute effects of EECP on the lower extremity hemodynamics. Studies have reported that inflation of the EECP-3 cuffs creates a high-pressure retrograde blood flow in the femoral arteries (Gurovich and Braith, 2013). Cai *et al.* also found that EECP could improve the blood circulation in lower extremities by combining animal and human experiments (Cai et al., 2000). In addition, our previous study showed that EECP increases FR of femoral artery (Zhang et al., 2021a). Meanwhile, Avery *et al.* found that EECP significantly increased FR and improved endothelium-dependent vasodilation in calf resistance arteries. EECP may provide a kind of “massage” on the peripheral function, starting to the elastic conduit arteries, the muscular conduit arteries, and extending to the skeletal muscle resistance arteries (Avery et al., 2014). The diastolic inflation/systolic deflation sequence of EECP-3 results in diastolic augmentation/systolic unloading and, leading to increased blood flow (Ozawa et al., 2001; Zhang et al., 2007).

However, our results are inconsistent, at least in part with above findings. After changing the limb cuffs, FR in the posterior tibial artery was significantly decreased both during EECP-2 and EECP-1. Werner *et al.* found that FR of the posterior tibial artery decreased to 69%±23% during EECP (Werner et al., 2007). Studies found that reduced peripheral flow during EECP may have similar physiologic effects like exercise in patients with symptomatic PAD. More importantly, it is contributed by changes of MV and PI (Werner et al., 2007). PI of posterior tibial and dorsalis pedis arteries markedly increased compared with baseline. In addition, MV of anterior tibial artery during EECP-3 was markedly higher than that at baseline (P=0.004), during EECP-1 (P=0.012) and EECP-2 ((P=0.012), while MV of dorsalis pedis artery during EECP-2 was markedly lower than that during EECP-3 (P=0.001) and EECP-1 (P=0.045). Werner *et al.* assessed blood flow velocity of the posterior tibial artery during EECP (Werner et al., 2007). They found significant increase in the MV and PI, showing a marked increase in retrograde blood flow. EECP creates a second diastolic pulse wave in all arterial vessels (Werner et al., 1999).

In this study, during EECP-2, PI and MV of dorsalis pedis artery were significantly increased and decreased, respectively. In addition, PI of dorsalis pedis artery was significantly higher in dorsalis pedis artery than that of other arteries during EECP-2. Findings of the present study support previous study that PI of the posterior tibial artery showed a 4-fold increase (Werner et al., 2007). Studies have been reported that PI is an important indicator of peripheral resistance (Gosling et al., 1971; Aleksic et al., 2004; Liang, 2020), which can be associated with postural changes, physiologic fluctuations and vascular disease (Delis et al., 2000; Ascher et al., 2007; Villar and Hughson, 2013). A second diastolic pulse wave in lower extremity artery was created by EECP-2. What’s more, changes of peripheral flow pattern during EECP-2 were also characterized by elevating PI.

Studies have also reported that regulation of FR is associated with vascular diameters and blood flow velocity (Levenson et al., 1985). In this study, blood flow velocity, like PSV and EDV, in the inferior knee artery significantly increased. Few studies investigated PSV of inferior knee artery during EECP. Zhang *et al.* also found that vascular diameter and PSV were significantly increased after EECP intervention. Besides that, acceleration of systolic peak velocity also increased. A study had showed that ACC of systolic peak velocity in the lower limb is an effective marker of peripheral artery disease (Li, 2017; Peltokangas et al., 2019). Cai *et al.* observed a 1.2-fold increase in femoral artery retrograde blood flow velocity (Cai et al., 2000). Based on a porcine EECP model, Zhang *et al.* demonstrated that blood flow velocity and wall shear stress of peripheral artery elevated by 1.3- and 2.1-fold, respectively, during EECP (Zhang et al., 2007). Additionally, EECP increases flow pulsatility and shear stress (Werner et al., 2007). Gurovich *et al.* also found that EECP significantly increased retrograde shear stress and retrograde-turbulent FR in the femoral artery (Gurovich and Braith, 2013). The mechanism responsible for this phenomenon is increased endothelial shear stress, which led to vascular anti-inflammatory changes in human umbilical vein endothelial cells (Duchene et al., 2009).

Moreover, they showed that changes of shear rate led by femoral artery vascular tone elicit an increased femoral baseline diameter after EECP intervention (Gurovich and Braith, 2013). Werner *et al.* reported that diameter in the posterior tibial artery were significantly decreased after EECP intervention (Werner et al., 2007). Sonka *et al.* demonstrated that femoral peak diameters were regarded as the single peak diameter investigated during the plateau phase after cuff deflation (Sonka et al., 2002). Dopheide *et al.* found that femoral artery diameter and vascular shear stress were significantly increased after supervised exercise training (Dopheide et al., 2017). Nevertheless, in the present study, Area of posterior tibial artery were significantly reduced during EECP-1, while there was no significant difference in Area during EECP-3 and EECP-2. Studies have reported that both flow- and pressure-induced forces playing an important role on vessel wall diameter (Helisch and Schaper, 2003; Schaper and Scholz, 2003; Heil and Schaper, 2004). Decreased Area induced by EECP-1 may be associated with different changes of pressure in both legs.

**Limitations**

Some limitations of this study should be emphasized. Firstly, in order to explore physiological changes of lower limb hemodynamics, participants in this study are healthy, young individuals, whereas EECP is normally prescribed for patients with cardiovascular disease. Secondly, we just investigate acute effects of EECP due to obtain each immediate response of hemodynamics. Finally, we did not measure lower limb-FMD, and these were not able to assess endothelial function after EECP in these arteries.

**Future direction**

Further studies investigating EECP with different sequence levels induced lower limb hemodynamics in patients with PAD are appropriate. In addition, long-term effects of EECP with different sequence levels on the lower limb vascular function in patients with PAD will be explored in the future.

## Conclusion

This study demonstrated that sensitive parameters in the anterior tibial artery, dorsalis pedis artery and posterior tibial artery are highlighted during EECP-3, EECP-2 and EECP-1, respectively. EECP-3 produces immediate improvement in blood flow, blood flow velocity and ACCs of anterior tibial artery. EECP-1 mainly regulated the hemodynamic indexes of posterior tibial artery, including FR and Area. By contrast, EECP-2 significantly regulated the PI and MV of dorsalis pedis artery. This present study will be beneficial to realize the personalized and precise treatment of PAD with external counterpulsation. EECP-3 may be recommended for patients with anterior tibial artery stenosis. EECP-2 may be recommended to improve lower arteries hemodynamics of patients with dorsal foot artery stenosis. On the contrary, EECP-2 and EECP-1 may be not suitable for treatment of posterior tibial artery.

# Conflict of Interest

The authors declared no competing interests.

# Author Contributions

YZ, CZ and GW proposed the scientific problems. YZ, YZ, and CZ designed the experiments. YZ, YZ, WW, YW, XX and JJ collected the experimental data. YZ and XZ processed and calculated the data. YZ conducted statistical analysis and wrote the draft manuscript. CZ and GW contributed to the revision and final version of manuscript.

# Funding

This work was, in part, supported by the National Key Research and Development Program of China (No.2020YFC2004400), National Natural Science Foundation of China [Grant No. 819770367 and 81670417]. Part of this research was supported by Shenzhen Key Clinical Discipline Funds (ZDXKJF-01002).

# References

Aleksic, M., Heckenkamp, J., Gawenda, M., and Brunkwall, J. (2004). Pulsatility index determination by flowmeter measurement: a new indicator for vascular resistance? *Eur Surg Res* 36(6)**,** 345-349. doi: 10.1159/000081642.

Ascher, E., Hingorani, A.P., and Marks, N.A. (2007). Popliteal artery volume flow measurement: a new and reliable predictor of early patency after infrainguinal balloon angioplasty and subintimal dissection. *J Vasc Surg* 45(1)**,** 17-23; discussion 23-14. doi: 10.1016/j.jvs.2006.09.042.

Avery, J.C., Beck, D.T., Casey, D.P., Sardina, P.D., and Braith, R.W. (2014). Enhanced external counterpulsation improves peripheral resistance artery blood flow in patients with coronary artery disease. *Appl Physiol Nutr Metab* 39(3)**,** 405-408. doi: 10.1139/apnm-2013-0309.

Braith, R.W., Conti, C.R., Nichols, W.W., Choi, C.Y., Khuddus, M.A., Beck, D.T., et al. (2010a). Enhanced External Counterpulsation Improves Peripheral Artery Flow-Mediated Dilation in Patients With Chronic Angina. *Circulation* 122(16)**,** 1612-1620. doi: 10.1161/circulationaha.109.923482.

Braith, R.W., Conti, C.R., Nichols, W.W., Choi, C.Y., Khuddus, M.A., Beck, D.T., et al. (2010b). Enhanced external counterpulsation improves peripheral artery flow-mediated dilation in patients with chronic angina: a randomized sham-controlled study. *Circulation* 122(16)**,** 1612-1620. doi: 10.1161/circulationaha.109.923482.

Buschmann, E.E., Brix, M., Li, L., Doreen, J., Zietzer, A., Li, M., et al. (2016). Adaptation of external counterpulsation based on individual shear rate therapy improves endothelial function and claudication distance in peripheral artery disease. *Vasa* 45(4)**,** 317-324. doi: 10.1024/0301-1526/a000544.

Buschmann, I., Pries, A., Styp-Rekowska, B., Hillmeister, P., Loufrani, L., Henrion, D., et al. (2010). Pulsatile shear and Gja5 modulate arterial identity and remodeling events during flow-driven arteriogenesis. *Development* 137(13)**,** 2187-2196. doi: 10.1242/dev.045351.

Caceres, J., Atal, P., Arora, R., and Yee, D. (2021). Enhanced external counterpulsation: A unique treatment for the "No-Option" refractory angina patient. *J Clin Pharm Ther* 46(2), 295-303. doi: 10.1111/jcpt.13330.

Cai, D., Wu, R., and Shao, Y. (2000). Experimental study of the effect of external counterpulsation on blood circulation in the lower extremities. *Clin Invest Med* 23(4)**,** 239-247.

Delis, K.T., Nicolaides, A.N., and Stansby, G. (2000). Effect of posture on popliteal artery hemodynamics. *Arch Surg* 135(3)**,** 265-269. doi: 10.1001/archsurg.135.3.265.

Dockery, F., Rajkumar, C., Bulpitt, C.J., Hall, R.J., and Bagger, J.P. (2004). Enhanced external counterpulsation does not alter arterial stiffness in patients with angina. *Clin Cardiol* 27(12)**,** 689-692.

Dopheide, J.F., Rubrech, J., Trumpp, A., Geissler, P., Zeller, G.C., Schnorbus, B., et al. (2017). Supervised exercise training in peripheral arterial disease increases vascular shear stress and profunda femoral artery diameter. *Eur J Prev Cardiol* 24(2)**,** 178-191. doi: 10.1177/2047487316665231.

Duchene, J., Cayla, C., Vessillier, S., Scotland, R., Yamashiro, K., Lecomte, F., et al. (2009). Laminar shear stress regulates endothelial kinin B1 receptor expression and function: potential implication in atherogenesis. *Arterioscler Thromb Vasc Biol* 29(11)**,** 1757-1763. doi: 10.1161/atvbaha.109.191775.

Fihn, S.D., Gardin, J.M., Abrams, J., Berra, K., Blankenship, J.C., Dallas, A.P., et al. (2012). 2012 ACCF/AHA/ACP/AATS/PCNA/SCAI/STS Guideline for the diagnosis and management of patients with stable ischemic heart disease: a report of the American College of Cardiology Foundation/American Heart Association Task Force on Practice Guidelines, and the American College of Physicians, American Association for Thoracic Surgery, Preventive Cardiovascular Nurses Association, Society for Cardiovascular Angiography and Interventions, and Society of Thoracic Surgeons. *J Am Coll Cardiol* 60(24)**,** e44-e164. doi: 10.1016/j.jacc.2012.07.013.

Gosling, R.G., Dunbar, G., King, D.H., Newman, D.L., Side, C.D., Woodcock, J.P., et al. (1971). The quantitative analysis of occlusive peripheral arterial disease by a non-intrusive ultrasonic technique. *Angiology* 22(1)**,** 52-55. doi: 10.1177/000331977102200109.

Gurovich, A.N., and Braith, R.W. (2013). Enhanced external counterpulsation creates acute blood flow patterns responsible for improved flow-mediated dilation in humans. *Hypertens Res* 36(4)**,** 297-305. doi: 10.1038/hr.2012.169.

Hashemi, M., Hoseinbalam, M., and Khazaei, M. (2008). Long-term effect of enhanced external counterpulsation on endothelial function in the patients with intractable angina. *Heart Lung Circ* 17(5)**,** 383-387. doi: 10.1016/j.hlc.2008.02.001.

Heil, M., and Schaper, W. (2004). Influence of mechanical, cellular, and molecular factors on collateral artery growth (arteriogenesis). *Circ Res* 95(5)**,** 449-458. doi: 10.1161/01.res.0000141145.78900.44.

Helisch, A., and Schaper, W. (2003). Arteriogenesis: the development and growth of collateral arteries. *Microcirculation* 10(1)**,** 83-97. doi: 10.1038/sj.mn.7800173.

Levenson, J.A., Simon, A.C., and Safar, M.E. (1985). Vasodilatation of small and large arteries in hypertension. *J Cardiovasc Pharmacol* 7 Suppl 2**,** S115-120.

Li, L. (2017). Measuring fluid shear stress with a novel Doppler-derived relative pulse slope index and maximal systolic acceleration approach to detect peripheral arterial disease and to modulate arteriogenesis [D] Dissertationen Charité.

Liang, H.L. (2020). Doppler Flow Measurement of Lower Extremity Arteries Adjusted by Pulsatility Index. *AJR Am J Roentgenol* 214(1)**,** 10-17. doi: 10.2214/ajr.19.21280.

Martin, J.S., Beck, D.T., and Braith, R.W. (2014). Peripheral resistance artery blood flow in subjects with abnormal glucose tolerance is improved following enhanced external counterpulsation therapy. *Appl Physiol Nutr Metab* 39(5)**,** 596-599. doi: 10.1139/apnm-2013-0497.

Masuda, D., Nohara, R., Hirai, T., Kataoka, K., Chen, L.G., Hosokawa, R., et al. (2001). Enhanced external counterpulsation improved myocardial perfusion and coronary flow reserve in patients with chronic stable angina; evaluation by(13)N-ammonia positron emission tomography. *Eur Heart J* 22(16)**,** 1451-1458. doi: 10.1053/euhj.2000.2545.

Montalescot, G., Sechtem, U., Achenbach, S., Andreotti, F., Arden, C., Budaj, A., et al. (2013). 2013 ESC guidelines on the management of stable coronary artery disease: the Task Force on the management of stable coronary artery disease of the European Society of Cardiology. *Eur Heart J* 34(38)**,** 2949-3003. doi: 10.1093/eurheartj/eht296.

Nichols, W.W., Estrada, J.C., Braith, R.W., Owens, K., and Conti, C.R. (2006). Enhanced external counterpulsation treatment improves arterial wall properties and wave reflection characteristics in patients with refractory angina. *J Am Coll Cardiol* 48(6)**,** 1208-1214. doi: 10.1016/j.jacc.2006.04.094.

Ozawa, E.T., Bottom, K.E., Xiao, X., and Kamm, R.D. (2001). Numerical simulation of enhanced external counterpulsation. *Ann Biomed Eng* 29(4)**,** 284-297. doi: 10.1114/1.1359448.

Peltokangas, M., Vakhitov, D., Suominen, V., Korhonen, J., Huotari, M., Verho, J., et al. (2019). Lower Limb Pulse Rise Time as a Marker of Peripheral Arterial Disease. *IEEE Trans Biomed Eng* 66(9)**,** 2596-2603. doi: 10.1109/tbme.2019.2892407.

Picard, F., Panagiotidou, P., Wolf-Putz, A., Buschmann, I., Buschmann, E., Steffen, M., et al. (2018). Usefulness of Individual Shear Rate Therapy, New Treatment Option for Patients With Symptomatic Coronary Artery Disease. *Am J Cardiol* 121(4)**,** 416-422. doi: 10.1016/j.amjcard.2017.11.004.

Picard, F., Panagiotidou, P., Wolf-Putz, A., Buschmann, I., Buschmann, E., Steffen, M., et al. (2020). Individual shear rate therapy (ISRT)-further development of external counterpulsation for decreasing blood pressure in patients with symptomatic coronary artery disease (CAD). *Hypertens Res* 43(3)**,** 186-196. doi: 10.1038/s41440-019-0380-x.

Schaper, W., and Scholz, D. (2003). Factors regulating arteriogenesis. *Arterioscler Thromb Vasc Biol* 23(7)**,** 1143-1151. doi: 10.1161/01.atv.0000069625.11230.96.

Sonka, M., Liang, W., and Lauer, R.M. (2002). Automated analysis of brachial ultrasound image sequences: early detection of cardiovascular disease via surrogates of endothelial function. *IEEE Trans Med Imaging* 21(10)**,** 1271-1279. doi: 10.1109/tmi.2002.806288.

Thakkar, B.V., Hirsch, A.T., Satran, D., Bart, B.A., Barsness, G., McCullough, P.A., et al. (2010). The efficacy and safety of enhanced external counterpulsation in patients with peripheral arterial disease. *Vasc Med* 15(1)**,** 15-20. doi: 10.1177/1358863X09106549.

Villar, R., and Hughson, R.L. (2013). Lower limb vascular conductance and resting popliteal blood flow during head-up and head-down postural challenges. *Clin Physiol Funct Imaging* 33(3)**,** 186-191. doi: 10.1111/cpf.12008.

Werner, D., Michalk, F., Hinz, B., Werner, U., Voigt, J.U., and Daniel, W.G. (2007). Impact of enhanced external counterpulsation on peripheral circulation. *Angiology* 58(2)**,** 185-190. doi: 10.1177/0003319707300013.

Werner, D., Schneider, M., Weise, M., Nonnast-Daniel, B., and Daniel, W.G. (1999). Pneumatic external counterpulsation: a new noninvasive method to improve organ perfusion. *Am J Cardiol* 84(8)**,** 950-952, a957-958. doi: 10.1016/s0002-9149(99)00477-4.

Wu, E., Desta, L., Broström, A., and Mårtensson, J. (2020). Effectiveness of Enhanced External Counterpulsation Treatment on Symptom Burden, Medication Profile, Physical Capacity, Cardiac Anxiety, and Health-Related Quality of Life in Patients With Refractory Angina Pectoris. *J Cardiovasc Nurs* 35(4)**,** 375-385. doi: 10.1097/jcn.0000000000000638.

Wu, E., Mårtensson, J., Desta, L., and Broström, A. (2021). Predictors of treatment benefits after enhanced external counterpulsation in patients with refractory angina pectoris. *Clin Cardiol* 44(2)**,** 160-167. doi: 10.1002/clc.23516.

Zhang, Y., Chen, Z., Zhouming, M., Zhou, W., Wang, H., Zhang, X., et al. (2021a). Acute Hemodynamic Responses to Enhanced External Counterpulsation in Patients with Coronary Artery Disease. *Front Cardiovasc Med***,** 1542.

Zhang, Y., He, X., Chen, X., Ma, H., Liu, D., Luo, J., et al. (2007). Enhanced external counterpulsation inhibits intimal hyperplasia by modifying shear stress responsive gene expression in hypercholesterolemic pigs. *Circulation* 116(5)**,** 526-534. doi: 10.1161/circulationaha.106.647248.

Zhang, Y., Mai, Z., Du, J., Zhou, W., Wei, W., Wang, H., et al. (2021). Acute Effect of Enhanced External Counterpulsation on the Carotid Hemodynamic Parameters in Patients With High Cardiovascular Risk Factors. *Front Physiol* 12**,** 615443. doi: 10.3389/fphys.2021.615443.

Zietzer, A., Buschmann, E.E., Janke, D., Li, L., Brix, M., Meyborg, H., et al. (2017). Acute physical exercise and long-term individual shear rate therapy increase telomerase activity in human peripheral blood mononuclear cells. *Acta Physiol (Oxf)* 220(2)**,** 251-262. doi: 10.1111/apha.12820.

**Figure legends**

Figure 1 The flowchart of whole experimental scheme

Figure 2 Ultrasound pictures and Doppler spectrum of anterior tibial artery at baseline, during EECP-3, EECP-1 and EECP-2 in order.

Figure 3. Significant changes of anterior tibial artery, posterior tibial artery and dorsalis pedis artery at baseline, during EECP-3, EECP-2 and EECP-1.

Note: a, significant differences between anterior tibial artery and posterior tibial artery; b, significant difference between dorsalis pedis artery and posterior tibial artery; c, significant differences between anterior tibial artery and dorsalis pedis artery

Figure 4. Effect of EECP-3, EECP-2 and EECP-1 on the flow rate (FR) of anterior tibial artery, posterior tibial artery and dorsalis pedis artery.

Figure 5. Effect of EECP-3, EECP-2 and EECP-1 on the Area of anterior tibial artery, posterior tibial artery and dorsalis pedis artery.

Figure 6. Effect of EECP-3, EECP-2 and EECP-1 on the pulsatility index (PI) of anterior tibial artery, posterior tibial artery and dorsalis pedis artery.

Figure 7. Effect of EECP-3, EECP-2 and EECP-1 on the peak systolic velocity (PSV) of anterior tibial artery, posterior tibial artery and dorsalis pedis artery.

Figure 8. Effect of EECP-3, EECP-2 and EECP-1 on the end-diastolic velocity (EDV) of anterior tibial artery, posterior tibial artery and dorsalis pedis artery.

Figure 9. Effect of EECP-3, EECP-2 and EECP-1 on the mean flow velocity (MV) of anterior tibial artery, posterior tibial artery and dorsalis pedis artery.

Figure 10. Effect of EECP-3, EECP-2 and EECP-1 on the systolic maximum acceleration (CCAs) of anterior tibial artery, posterior tibial artery and dorsalis pedis artery.

**Table 1 Basic characteristics of participants**

| **Characteristics** | **Subjects (n=20)** |
| --- | --- |
| Age, yr | 25.51±2.28 |
| Height, cm | 173.20±4.74 |
| Weight, kg | 66.83±7.38 |
| Smoking | 0 |
| Drinking | 0 |
| Exercise habits, n(%) | 6(30) |
| Sleep disorders, n(%) | 5(25) |
| Family history, n(%) | 3(15) |
| SBP (mmHg) | 118.80±7.92 |
| DBP (mmHg) | 72.35±4.52 |
| HR (bpm) | 64.75±8.93 |
