# Supplementary material for: Effects of Enhanced External Counterpulsation With Different Sequential Levels on Lower Extremity Hemodynamics
Source: Front Cardiovasc Med. 2021 Dec 24;8:795697. doi: 10.3389/fcvm.2021.795697 (PMC8739776; doi:10.3389/fcvm.2021.795697)
Supplement: Supplementary file 4 [file Table_4.DOCX]

**Response to Reviewers’ Comments**

**Manuscript ID:** 795697

**Manuscript Title:** Effects of Enhanced External Counterpulsation with Different Sequential Levels on Lower Extremity Hemodynamics

Dear Editor and Reviewers,

We thank you for the valuable comments and suggestions. We are grateful to the Editor for coordinating this review process. All comments have been addressed in the revised manuscript. In the following, our point-to-point responses to the comments are offered.

Sincerely,

Authors

**Response to Editor**

**Comment 1:**

Compare and contrast your study with others in the most relevant world literature, particularly the recent literature.

**Response:**

Thank you for your comments. The recent literature was added in the Introduction section.

**Comment 2:**

What new information is sufficient to modify existing clinical practice?

**Response:**

Thank you for your comments. Currently, few studies explore acute effects of EECP on peripheral hemodynamics. This study investigated acute hemodynamics of lower extremities during the Enhanced External Counterpulsation with three-level sequence at hips, thighs and calves (EECP-3), two-level sequence at hips and thighs (EECP-2) and single leg three-level sequence (EECP-1). It demonstrated that EECP-3, EECP-2 and EECP-1 induced different hemodynamic responses in the anterior tibial artery, dorsalis pedis artery and posterior tibial artery, respectively. More importantly, EECP-3 creates acute improvement in blood flow, blood flow velocity and ACCs of anterior tibial artery. In addition, EECP-1 and EECP-2 significantly increase blood flow velocity and peripheral resistance of inferior knee artery, whereas they markedly reduce blood flow in the posterior tibial artery. Further research is needed to examine the long-term effects of EECP on PAD patients with different lower limb stenosis.

**Comment 3:**

What are the conclusions and implications for current practice, and particularly for future research that may have a significant impact on clinical decisions?

**Response:**

Thank you so much for your suggestions. The conclusion section was rewritten. EECP-3 may be recommended for patients with anterior tibial artery stenosis. EECP-2 may be recommended to improve lower arteries hemodynamics of patients with dorsal foot artery stenosis. On the contrary, EECP-2 and EECP-1 may be not suitable for treatment of posterior tibial artery. Thus, these findings may have a significant impact on personalized and precise treatment of PAD with external counterpulsation. Further research is needed to verify long-term effects of EECP on PAD patients with different lower limb stenosis.

**Comment 4:**

What does this study add to the literature?

**Response:**

Thank you for your comments. This study investigated that EECP-3, EECP-2 and EECP-1 induced different hemodynamic responses in the anterior tibial artery, dorsalis pedis artery and posterior tibial artery, respectively. In this study, we found that EECP-3 creates acute improvement in blood flow, blood flow velocity and ACCs of anterior tibial artery. In addition, EECP-1 and EECP-2 significantly increase blood flow velocity and peripheral resistance of inferior knee artery, whereas they markedly reduce blood flow in the posterior tibial artery. It will be beneficial to realize the personalized and precise treatment of PAD with external counterpulsation.

**Comment 5:**

At the end of the Discussion, under the subheading "Limitations," review the limitations of your study.

**Response:**

Thank you for your comments. The subheading “Limitations” was added, and the contents of limitation were rewritten.

“Some limitations of this study should be emphasized. Firstly, in order to explore physiological changes of lower limb hemodynamics, participants in this study are healthy, young individuals, whereas EECP is normally prescribed for patients with cardiovascular disease. Secondly, we just investigate acute effects of EECP due to obtain each immediate response of hemodynamics. Finally, we did not measure lower limb-FMD, and these were not able to assess endothelial function after EECP in these arteries.”

**Comment 6:**

At the end of the limitations, under the subheading " Future directions".

**Response:**

Thank you so much for your suggestions. The subheading "Future directions" was added in this study.

“Future direction

Further studies investigating EECP with different sequence levels induced lower limb hemodynamics in patients with PAD are appropriate. In addition, long-term effects of EECP with different sequence levels on the lower limb vascular function in patients with PAD will be explored in the future.”

**Comment 7:**

Conclusion: Take special care to draw your conclusions only from your results and verify that your conclusions are firmly supported by your data

**Response:**

Thank you for your comments. The conclusion section was rewritten according to Review’s comment and suggestions.

“This study demonstrated that sensitive parameters in the anterior tibial artery, dorsalis pedis artery and posterior tibial artery are highlighted during EECP-3, EECP-2 and EECP-1, respectively. EECP-3 produces immediate improvement in blood flow, blood flow velocity and ACCs of anterior tibial artery. EECP-1 mainly regulated the hemodynamic indexes of posterior tibial artery, including FR and Area. By contrast, EECP-2 significantly regulated the PI and MV of dorsalis pedis artery. This present study will be beneficial to realize the personalized and precise treatment of PAD with external counterpulsation. EECP-3 may be recommended for patients with anterior tibial artery stenosis. EECP-2 may be recommended to improve lower arteries hemodynamics of patients with dorsal foot artery stenosis. On the contrary, EECP-2 and EECP-1 may be not suitable for treatment of posterior tibial artery.”

**Comment 8:**

References Update.

**Response:**

Thank you so much for your suggestions. Eight references were added.

[1] *Caceres, J., Atal, P., Arora, R., and Yee, D. (2021). Enhanced external counterpulsation: A unique treatment for the "No-Option" refractory angina patient. J Clin Pharm Ther 46(2), 295-303. doi: 10.1111/jcpt.13330.*
[2] *Ozawa, E.T., Bottom, K.E., Xiao, X., and Kamm, R.D. (2001). Numerical simulation of enhanced external counterpulsation. Ann Biomed Eng 29(4)****,*** *284-297. doi: 10.1114/1.1359448.*
[3] *Picard, F., Panagiotidou, P., Wolf-Putz, A., Buschmann, I., Buschmann, E., Steffen, M., et al. (2018). Usefulness of Individual Shear Rate Therapy, New Treatment Option for Patients With Symptomatic Coronary Artery Disease. Am J Cardiol 121(4)****,*** *416-422. doi: 10.1016/j.amjcard.2017.11.004.*
[4] *Picard, F., Panagiotidou, P., Wolf-Putz, A., Buschmann, I., Buschmann, E., Steffen, M., et al. (2020). Individual shear rate therapy (ISRT)-further development of external counterpulsation for decreasing blood pressure in patients with symptomatic coronary artery disease (CAD). Hypertens Res 43(3)****,*** *186-196. doi: 10.1038/s41440-019-0380-x.*
[5] *Wu, E., Desta, L., Broström, A., and Mårtensson, J. (2020). Effectiveness of Enhanced External Counterpulsation Treatment on Symptom Burden, Medication Profile, Physical Capacity, Cardiac Anxiety, and Health-Related Quality of Life in Patients With Refractory Angina Pectoris. J Cardiovasc Nurs 35(4)****,*** *375-385. doi: 10.1097/jcn.0000000000000638.*
[6] *Wu, E., Mårtensson, J., Desta, L., and Broström, A. (2021). Predictors of treatment benefits after enhanced external counterpulsation in patients with refractory angina pectoris. Clin Cardiol 44(2)****,*** *160-167. doi: 10.1002/clc.23516.*
[7] *Zhang, Y., Chen, Z., Zhouming, M., Zhou, W., Wang, H., Zhang, X., et al. (2021a). Acute Hemodynamic Responses to Enhanced External Counterpulsation in Patients with Coronary Artery Disease. Front Cardiovasc Med****,*** *1542.*
[8] *Zhang, Y., Mai, Z., Du, J., Zhou, W., Wei, W., Wang, H., et al. (2021). Acute Effect of Enhanced External Counterpulsation on the Carotid Hemodynamic Parameters in Patients With High Cardiovascular Risk Factors. Front Physiol 12****,*** *615443. doi: 10.3389/fphys.2021.615443.*

**Response to Reviewer #2**

**Comment 1:**

the manuscript is well designed but the most concerning point is in figure 2 i do not understand why there was no fixation of the maximum velocity scale in order to make a good judgment of many sentences without new relevant references.

**Response:**

Thank you so much for your suggestions. The clearest image is displayed in the Figure 2. The maximum velocity scale was not fixed because larger change of systolic peak velocity at baseline (70.79±16.45mL/min), during EECP-3 (131.76±52.18 mL/min), EECP-1 (96.80±23.17 mL/min) and EECP-2 (101.74±22.69 mL/min). In addition, Gurovich *et al.* also investigated EECP-induced blood flow pattern via ultrasonic blood flow spectrum. It was also no fixation of the maximum velocity scale in order to show a clear image [1].

[1] *Gurovich A N, Braith R W. Enhanced external counterpulsation creates acute blood flow patterns responsible for improved flow-mediated dilation in humans. Hypertension Research Official Journal of the Japanese Society of Hypertension, 2013, 36(4):297-305.*

**Comment 2:**

the result should be expressed in a more detailed action with more figures in order to focus on the main interesting points.

**Response:**

Thank you for your comments. Main interesting points have been reorganized and refined in the result section. In addition, Figure 3 was added to show continuous change trends of hemodynamics and differences among inferior knee arteries at baseline, during EECP-3, EECP-1 and EECP-2.

Figure 3. Significant changes of anterior tibial artery, posterior tibial artery and dorsalis pedis artery at baseline, during EECP-3, EECP-2 and EECP-1.

Note: a, significant differences between anterior tibial artery and posterior tibial artery; b, significant difference between dorsalis pedis artery and posterior tibial artery; c, significant differences between anterior tibial artery and dorsalis pedis artery

**Comment 3:**

discussion is so poor must be rearranged again

**Response:**

Thank you for your suggestions. The discussion section was rewritten according to Reviewer’s comments.

**Comment 4:**

results section must be sectioned into main titles findings

**Response:**

Thank you for your comments. The results section was revised as main titles findings.

“The baseline information, including age, gender, height, weight, risk factors (smoking, drinking, family history and sleep disorders, and exercise habits were shown as below at resting conditions before EECP intervention (Table 1).

Original ultrasonic pictures and Doppler spectrum of anterior tibial artery at baseline, during EECP-3, EECP-2 and EECP-1 were illustrated in Figure 2. Different hemodynamic responses (e.g. blood flow, blood flow velocity and PI) among the anterior tibial artery, posterior tibial artery and dorsalis pedis artery were showed in Figure 3. FR, Area and PSV in the anterior tibial artery were significantly higher than in other arteries (all P<0.01). MV of anterior tibial artery was also significantly higher than that of other arteries during EECP-3 and EECP-2 (both P<0.01). However, PI of dorsalis pedis artery was significantly higher in dorsalis pedis artery than that of other arteries during EECP-2 (both P<0.01).

Additionally, in order to clearly show hemodynamic response in each participant, effects of EECP on the hemodynamic variables varied in each subject were illustrated in Figures 4 to 10 and summarized in S1 Table 2.

**Blood flow**

FR of posterior tibial artery was significantly decreased during EECP-2 and EECP-1 compared with baseline (both P<0.05, Figure 4(a)). However, FR of anterior tibial artery was significantly increased during EECP-3 (P=0.048, Figure 4(b)). FR of dorsalis pedis artery during EECP-2 was higher than that during EECP-3. In addition, Area which is used to calculate FR in the posterior tibial artery during EECP-1 was significantly lower than that at baseline, during EECP-3 and EECP-2 (all P<0.01, Figure 5(a)). However, there was no significant difference in Area of anterior tibial artery and dorsalis pedis artery at baseline, during EECP-3, EECP-1 and EECP-2 (all P>0.05, Figure 5(b and c)).

**Pulsatility index**

PI of posterior tibial artery during EECP-3, EECP-2 and EECP-1 was significantly higher compared with baseline (all P<0.01, Figure 6(a)). PI of anterior tibial artery was also significantly higher during EECP-3 than that during EECP-1 (P=0.015) and EECP-2 (P=0.049). Moreover, PI of dorsalis pedis artery was markedly higher during EECP-2 compared with EECP-1 (P=0.003, Figure 6(c)).

**Blood flow velocity**

PSV in the inferior knee artery was significantly increased during EECP-3, EECP-2 and EECP-1 compared with baseline (all P<0.01, Figure 7). In addition, PSV of anterior tibial artery was significantly higher during EECP-3 than that during EECP-1 (P=0.002) and EECP-2 (P=0.005).

Compared with baseline, EDV of inferior knee artery was significantly increased during EECP-3, EECP-2 and EECP-1 (all P<0.01, Figure 8). EDV of posterior tibial artery and dorsalis pedis artery during EECP-1 was higher than that during EECP-3 (both P<0.01, Figure 8(a)). Additionally, EDV of anterior tibial artery and posterior tibial artery was also significantly higher during EECP-2 than that during EECP-3 in this study (both P<0.01, Figure 8(a and b)).

MV of anterior tibial artery during EECP-3 was markedly higher than that at baseline (P=0.004), during EECP-1 (P=0.012) and EECP-2 ((P=0.012), Figure 9), while MV of dorsalis pedis artery was markedly lower during EECP-2 than that during EECP-3 (P=0.001) and EECP-1 (P=0.045, Figure 9(c)). There was no significantly change of MV in the posterior tibial artery at each stage (all P>0.05, Figure 9).

**Systolic maximum acceleration (CCAs)**

During EECP-3, CCAs in the anterior tibial artery, posterior tibial artery and dorsalis pedis artery was significantly increased compared with baseline (all P<0.01, Figure 10). However, there was no significant difference in CCAs at baseline, during EECP-1 and EECP-2 (all P>0.05).”

**Comment 5:**

the last paragraph with limitation is very bad written.

**Response:**

Thank you so much for your suggestions. The part of limitation was rewritten according to Reviewer’s comments.

“**Limitations**

Some limitations of this study should be emphasized. Firstly, in order to explore physiological changes of lower limb hemodynamics, participants in this study are healthy, young individuals, whereas EECP is normally prescribed for patients with cardiovascular disease. Secondly, we just investigate acute effects of EECP due to obtain each immediate response of hemodynamics. Finally, we did not measure lower limb-FMD, and these were not able to assess endothelial function after EECP in these arteries.”

**Comment 6:**

I suggest the certificated English editing to improve the paper quality.

**Response:**

Thank you so much for your suggestions. The language expression and grammar were carefully revised in this manuscript.

**Response to Reviewer #1**

**Comment 1:**

think more detail is needed as to the actual benefit of this test / investigation. also this was performed in 20 healthy patients with no vascular disease. if applied to pad patients would this only benefit those with anterior tibial artery stensoses as stated in the conclusion.

**Response:**

Thank you for your comments. We found that EECP-3 creates acute improvement in blood flow, blood flow velocity and ACCs of anterior tibial artery. In addition, EECP-1 and EECP-2 significantly increase blood flow velocity and peripheral resistance of inferior knee artery, whereas they markedly reduce blood flow in the posterior tibial artery. It will be beneficial to realize the personalized and precise treatment of PAD with external counterpulsation. EECP-3 may be recommended for patients with anterior tibial artery stenosis. EECP-2 may be recommended to improve lower arteries hemodynamics of patients with dorsal foot artery stenosis. On the contrary, EECP-2 and EECP-1 may be not suitable for treatment of posterior tibial artery.

**Comment 2:**

also firm conclusions are needed as to the way forward and if it does have a place in what group of patients and for how long. A clearer picture would be nice as to the location of the EECP.

**Response:**

Thank you so much for your suggestions. This study just investigated acute hemodynamics of lower extremities during the Enhanced External Counterpulsation with three-level sequence at hips, thighs and calves (EECP-3), two-level sequence at hips and thighs (EECP-2) and single leg three-level sequence (EECP-1). Thus, current findings cannot provide a clearer picture showing location of the EECP. Further research is needed to examine the long-term effects of EECP on PAD patients with different limb arteriostenosis.

**Comment 3:**

this is well written but an=m uncertain as to the long term benefits for PAD patients particularly as this has been performed in normal healthy patients.

**Response:**

Thank you so much for your suggestions. Further research is needed to examine the long-term effects of EECP on PAD patients with different limb arteriostenosis.

**Comment 4:**

maybe a pilot study should be incorporated with pad patients as an early results section.

**Response:**

Thank you for your comments. Based on the current findings, we have collected ten patients with arteriosclerosis obliterans to conduct long-term treatment effects of EECP. We will perform a detailed scheme for further study according to Editor and Reviewers’ comments and suggestions.

**List of change**

1. The result and conclusion part were modified in the abstract section;
2. The recent literature has been added in the introduction section;
3. Figure 2 was modified according to Reviewer’s comments;
4. Result section was rearranged and refined according to Editor and Reviewers ‘comments;
5. Discussion section was rewritten according to Reviewers’ comments;
6. Eight references were added in the discussion section;
7. All the formats, language expression and grammar were revised in this study.

**************This is the end of the document************
